# The role of patients, caregivers, and communities in Learning Health Systems: a narrative review

**DOI:** 10.3389/frhs.2025.1606124

**Published:** 2025-10-21

**Authors:** Rachel Giacomantonio, Susan Dunn, Donna Rubenstein, Mari Somerville, Kristen Hemming, Lauren McLaughlin, Teresa Finlay, Joseph Wherton

**Affiliations:** ^1^ Nuffield Department of Primary Care Health Sciences, University of Oxford, Oxford, United Kingdom; ^2^ Maritime SPOR SUPPORT Unit (MSSU), Halifax, NS, Canada; ^3^ Nova Scotia Health (NSH), Halifax, NS, Canada; ^4^ Department of Health, Dalhousie University, Halifax, NS, Canada; ^5^ IWK Health, Halifax, NS, Canada

**Keywords:** engagement, community, patients, caregivers, learning health systems, patient-oriented research

## Abstract

Learning Health Systems (LHSs) seek to continuously generate and apply evidence in clinical practice. Most Canadian LHS models emphasize engagement with patients, caregivers, and communities (herein contributors). Yet, there is limited guidance about how engagement works in these dynamic systems and how it may differ from engagement in other settings, for example patient-oriented research and quality improvement. This review examines engagement activities in existing and emerging LHSs for insights into the roles that contributors play in creating patient-oriented and equitable LHSs. A narrative review was conducted using the PerSPEcTiF framework. Search terms were identified for three domains: contributors, LHSs operating in direct patient care settings, and active engagement. Four databases (PubMed-MEDLINE, CINAHL, PsycINFO, Embase) were searched in December 2022. Articles were screened using a domain-based rubric and sampled for richness. Data were extracted including who was engaged, when, where, and how. Engagement activities were coded inductively, then deductively using the International Association for Public Participation (IAP2) Spectrum of Public Participation. An advisory group, including a Patient Partner, provided input at several stages in the project. Thirty-six articles describing engagement in 30 LHSs were included. In all, 192 engagement activities were coded to create a taxonomy of engagement; 139 activities were also coded to the IAP2 Spectrum. Contributors' influence over decision-making was often unclear or limited, with engagement frequently occurring after LHS implementation. However, LHSs also provided contributors with opportunities to engage in deliberative system design and effect change through distributed leadership. Ten contributor roles were synthesized, serving three functions: “System Shapers” (designing and defining LHSs), “Community and Capacity Builders” (expanding and supporting LHSs), and “Implementers” (hands-on efforts). This review provides an overview of engagement in LHSs, demonstrating how these practices can both build on and be constrained by engagement traditions in patient-oriented research and quality improvement. Findings offer a starting point for designing meaningful contributor roles and highlight opportunities to reimagine engagement practices by embedding contributors within systems and engaging communities beyond patient care settings.

## 1 Introduction

Learning Health Systems (LHSs) are flexible models of care that seek an ever-closer union between healthcare, research, and quality improvement, treating the provision of healthcare as a means of generating and applying evidence quickly in medical practice (1). There is no single definition of LHSs (2) but, essentially, these healthcare systems are designed “to continuously, routinely, and efficiently study and improve themselves” [(3), p. 1], through a “virtuous cycle of health improvement” [(1), p. 45]. LHS models often call for engagement with patients, caregivers, and communities (herein contributors) [e.g., (4, 5)], but effective engagement requires better understanding both of how LHSs work in practice, and how best to engage these important contributors within these models.

First proposed by the now National Academy of Medicine in 2007 (6),^1^ LHSs emerged at a time of intense optimism about the transformative power of technology within healthcare (7). Under this model, medical care is seen as a way to generate data to improve quality of care, produce innovative treatments, and lower costs (8)—a data-driven vision for achieving the “quadruple aim” of improving patient and provider experience, bettering population health, and reducing healthcare expenditures (4). As a result, patients often feature in LHS literature as either sources of routine data [e.g., (9)] or ready participants for research [e.g., (10)], including examining the necessity (or not) of their consenting to various uses of their healthcare information [e.g., (11–13)]. By extension, considerable effort has focused on the potential for health data to support an active role for patients in managing their health [e.g., (14)]. This narrow focus on patients as “donors’ of data” [(15), p. 104] or recipients of evidence-driven insights into their own care may limit opportunities to engage patients in LHS design and implementation (15, 16), with few functioning LHSs reporting aspects of patient-oriented research (17).

There is a growing interest in LHSs in Canada, which is influenced by several factors including the potential to harness institutional synergy amongst Canadian health system actors including funders, policy-makers, and academics (4), and the desire to use rapid learning and adaptation to respond to rising pressures on publicly-funded provincial health systems—a process arguably accelerated by the COVID-19 pandemic (18, 19). Canadian LHS models emphasize an active role for patients and/or connections to community beyond direct patient care settings [e.g., (4, 5)]. Lavis et al. (5) identify “engaged patients” as one of seven core characteristics of a LHS. Similarly, Menear et al. (4) argue that a “transformative feature of LHSs is their emphasis on patient and community engagement” (p. 5), which fits within the “Social” pillar of their model. Thus, attempts at LHS implementation in Canada tend to recognize engagement as ensuring that LHSs are “anchored on patient needs, perspectives and aspirations” [(18), p. 8].

In Canada, LHSs are also being designed within an established and evolving culture of patient engagement in health research (20), largely driven by the Canadian Institutes of Health Research (CIHR) Strategy for Patient-Oriented Research (SPOR). First introduced in 2011, this national policy framework supports active and meaningful engagement of patients as partners in all aspects of health research (21). Patient-oriented research (POR) is distinct but related to other engagement traditions, for example patient-centered outcomes research in the United States (US) [e.g., (22)] and Patient and Public Involvement in the United Kingdom (UK) [e.g., (23, 24)]. Akin to approaches Madden and Speed (25) describe as “pragmatic and outcome orientated” (p. 3), POR aims to embed lived experience in applied research by connecting patients with other health system stakeholders,^2^ early and often, to improve the relevance of research and speed its application in practice (21).

In 2022, the CIHR, Canadian provincial governments and partners jointly announced more than $250M CDN of additional funding for SPOR SUPPORT Units (27–34), with the stipulation that LHSs become a core component of each Unit's work (35). This closely tied patient engagement to LHSs and provided resources for supporting LHS implementation within and across Canadian jurisdictions. Several SPOR SUPPORT Units have created LHS models for their contexts (36–38). Building on POR traditions, these interpretations also emphasize an active role for contributors and prioritize health equity (36–38).

Despite these favourable conditions—an appetite for LHSs, availability of resources, a tradition of patient engagement, and the conceptual importance placed on engagement—there remain gaps in understanding about how best to engage patients in LHSs such that engagement is also seen as a barrier to LHS implementation (4, 17). Even while affirming its importance, Menear et al. (4) acknowledge that there is limited guidance about how to adapt existing engagement practices specifically within LHSs, including “few mechanisms…to ensure strong patient involvement in health system design and priority setting” (p. 9). Others have noted lack of guidance and limited roles as persistent hurdles to meaningful engagement within LHSs (16, 17). Thus, engagement reflects a broader “gap between the promise and practice of LHSs” [(39), p. 4], whereby a largely theoretical literature describes components of LHSs without a clear description of how to best implement them (17). This creates a two-fold challenge, first to understand how the LHS operates, and then how best to engage contributors within this context. This narrative review seeks to close this gap by collating and critically analysing examples of engagement activities within LHSs, examining contextual factors that impact engagement, and describing the roles that contributors play within various LHSs, with particular attention to how they may differ from engagement in patient-oriented research.

## 2 Methods

This review explores a variable and subjective phenomenon (engagement) as it occurs within complex systems (LHSs)—a combination that makes for a highly heterogenous literature that is not amenable to rigid systematic review methods characterized by pre-defined research questions and strict protocols (40). Instead, this narrative review draws on Boell and Cecez-Kecmanovic's hermeneutic method (41), a flexible approach that focuses on interpretative understanding developed through progressive engagement with the literature (40, 41). This study was undertaken in Nova Scotia, Canada. Handwritten reflexive journals, maintained by RG, provided a structured way to iteratively explore the literature (e.g., noting common themes, key questions) and increased awareness of her positionality, for example how past experiences as a patient and current work in patient-oriented research influenced the analysis, as well as how personal privilege creates blind spots towards inequities (42). Key steps are summarized below; activities were often iterative and overlapping. All steps were performed by a student lead (RG), with input from supervisors (JW, TF), and a Stakeholder Advisory Group (DR, MS, SD, LM, KH).

### 2.1 Stakeholder engagement

A Stakeholder Advisory Group (SAG) was created to seek specific perspectives, identify gaps in local knowledge, avoid duplication of efforts, and connect findings to practice (21, 23, 43, 44). Recruitment was purposive and limited to five members, including a patient partner, engagement staff, and an LHS researcher. All members were offered an honorarium. Prior to convening, all members completed a short online survey in Survey Monkey (45) to learn about their experience and interest in the topic. Survey results informed the agenda for a virtual meeting held in November 2022, where SAG members helped refine the research question and revise a list of search terms. This session informed the search strategy, which was emailed to all SAG members for review. Preliminary findings were also presented to two SAG members (DR, SD) in April 2023, when a “member reflections” exercise was performed. Stakeholder engagement and its impact are reported throughout and summarized in Supplementary Material S1 using the Guidance for Reporting Involvement of Patients and the Public short form (GRIPP2-SF) standard template (46).

### 2.2 Literature searches

An initial search was performed in October 2022. Both PROSPERO and PubMed-MEDLINE were searched for literature reviews (of any type) on engagement in LHSs (Supplementary Material S2). None were found, although several covered engagement in other settings [e.g., (47–51)]. While narrative review does not require it (40, 52), a more structured search strategy was developed to create an entry point into the variable LHS literature. The search strategy, summarized below, was reviewed by two health librarians.

#### 2.2.1 Selecting search terms and defining concepts

The core search strategy hinges on three core concepts—contributors, engagement practices, and LHSs—each of which is described by a range of terms. Many of these terms have overlapping meanings, subtle context-specific nuances (2, 53), and/or politicized interpretations (54, 55). Diversity of terms can lead to unclear meaning and fragmentation in the literature (56, 57). Defining terms and identifying potential synonyms increases clarity and consistency, and enables a more comprehensive search strategy (58). Drawing on prior knowledge, a list of search terms was drafted then revised based on the literature on patient engagement (47, 49, 50) and LHSs (2, 59), as well as review protocols on engagement-related topics (58, 60, 61). This list was refined with feedback from the SAG. Notably, SAG members pointed to established, if siloed and distinct, traditions of engagement within research and quality improvement. To increase the relevance of search results, the setting was restricted to terms specific to learning health systems, organizations, and/or networks. This approach is consistent with previous research (17, 59), while also allowing some flexibility to capture different approaches to LHS implementation at the meso- and macro-level. Advocacy-related terms were excluded, as the SAG did not see this as a core function of engagement within local health systems. Finally, taking a similar approach to Chrysikou et al. (58), terms were grouped into “domains of terms.” Domains of terms were mapped against the first three components of the PerSPEcTiF question framework: perspective (Per), setting (S), phenomenon of interest (P), environment (E), comparison (c), timing (Ti) and findings (F) (Table 1). This question framework accounts for context in complex interventions (62), and provided structure for subsequent data extraction and analysis.

**Table 1:** Domains of terms and definitions mapped to the PerSPEcTiF question framework.

PerSPEcTiF question framework	Terms	Definition for this study	Additional search terms
Perspective (Per)	Domain: Contributors – Any patients, caregivers, communities and/or publics.		
	Patients and caregivers	“An overarching term that includes individuals with personal experience of a health issue and informal caregivers, including family and friends.” [(21), p. 5]	Patient Partner(s) Patient and Family Advisor(s) (PFAs) Patient/Public Partner(s) Patient Experience Advisor** People with Lived Experience (PwLE) Experts by experience* Consumers Client** Users Advisors Lay person Caregivers Family Relatives* Essential Care Partner**
	Community/Communities	“A group of people with diverse characteristics who are linked by social ties, share common perspectives, and engage in joint action.” [(134), p. 1929]	Population(s) Equity-Seeking Groups Parties** Interested parties**
	Public(s)	“The people as a whole.” (135)	Citizens Populace
Setting (S)	Domain: Learning Health Systems – Limited to health systems that self-describe as LHSs using one or more key terms and where direct patient care is provided.		
	Learning Health System (LHS)	“Dynamic health ecosystems where scientific, social, technological, policy, legal and ethical dimensions are synergistically aligned to enable cycles of continuous learning and improvement to be routinised and embedded across the system, thus enhancing value through an optimised balance of impacts on patient and provider experience, population health and health system costs.” [(4), p. 3]	Learning Healthcare System Learning Health Organization Integrated Learning Health System Learning Health Networks Clinical Learning Networks Rapid Learning System
Phenomenon of Interest (P)	Domain: Engagement – Any form of active engagement of contributors, in line with the CIHR definition (21).		
	Patient engagement	“Meaningful and active collaboration in governance, priority setting, conducting research and knowledge translation. Depending on the context, patient-oriented research may also engage people who bring the collective voice of specific, affected communities.” [(21), p. 5]	Patient Participation (MeSH term) Patient Partnership Patient and Public Engagement (PPE) Patient and Public Involvement (PPI) Patient/public involvement and engagement (PPIE)* Patient and Family Engagement (PFE)* Consumer Involvement Consumer Engagement* Public Participation Citizen Participation Citizen Engagement Community Engagement (CE)* Patient-driven research** Patient-led research** Participatory Action Research Community-Based Participatory Research Co-design Co-production Evidence-based co-design (EBCD)*
Environment (E)	Healthcare facilities of any type (e.g., hospitals, community clinics, academic medical centres, etc.), in any location (e.g., urban or rural, any country), and serving any population (e.g., any age, health condition, etc.)		
Comparison (c) (*optional*)	None		
Time (Ti)	When engagement first occurs relative to LHS implementation		
Findings (F)	Findings about the role(s) played by contributors within LHSs		

An initial list of terms was created based on prior knowledge and revised based on initial searches (*) and in consultation with stakeholders (**). Where possible, definitions were selected from within Canadian health research and care context.

#### 2.2.2 Searches, screening, and sampling

Searches were performed for the terms included in each core domain—Perspective (contributors), Setting (LHSs in direct patient care settings), and Phenomenon (active engagement)—and were limited to the title, abstract and keywords. Where available, Medical Subject Headings (MeSH) terms were included. The initial search strategy was developed for PubMed-MEDLINE (Supplementary Material S2), and adapted for CINAHL, PsycINFO, and Embase. Databases were searched in December 2022. Search syntax translations and search results were tracked in Microsoft Excel (63) (Supplementary Material S3). Targeted keyword searches were used to hand search highly-relevant journals: *Learning Health Systems*, *Health Expectations, Research Involvement and Engagement, Health Research Policy and Systems*, and *BMC Health Services Research*. Additionally, MS shared a collection of peer-reviewed literature that was tagged for patient engagement in another LHS review (17, 59). To maintain a manageable sample, all grey literature was excluded.

The core search results, as well as peer-reviewed literature recommended by MS and/or identified during the initial search, were uploaded to Covidence, a web-based software platform, for deduplication and screening (64). Screening was complicated by the heterogeneity of the literature and, as a result, inclusion and exclusion criteria were refined through three rounds of title and abstract screening. To speed the process, studies likely to meet inclusion criteria were tagged “highly relevant” and moved directly to full-text screening. Gradually, through this familiarization process, a domain-based screening rubric was developed (Table 2).

**Table 2:** Domain-based screening rubric.

Inclusion criteria	Exclusion criteria
Sources were included if they satisfied all the following criteria: **Setting** • LHS described using domain terms (Table 1) • Direct patient care is provided within LHS and/or source provides examples from LHS(s) providing direct patient care **Perspectives** • Patients, caregivers, communities and/or publics **Phenomenon** • Active engagement in line with the CIHR definition for patient engagement (Table 1) **Pragmatic inclusions** • Literature from academic journals • English language	Sources were excluded if they were classified as any of the following: **Irrelevant** • Not LHSs (e.g., London Handicap Scale, Length of Hospital Stay) **Wrong setting** • Not self-described LHS (e.g., engagement but not in LHS) • LHS but not direct patient care (e.g., conceptual papers, peer-support platforms, regulatory/policy settings, etc.) • “LHS light” (e.g., research described as consistent with LHS approach) **Wrong perspective** • Only clinicians or other non-contributors • No contributor perspectives (e.g., data-driven technical studies) **Wrong phenomenon** • Contributors treated only as passive recipients of healthcare or data sources • Engagement does not include contributors (e.g., professional communities of practice not including contributors) • Vague or undefined recommendations to engage patients/publics • Patient participation solely in their own care **Pragmatic exclusions** • Wrong source type (e.g., conference abstracts, posters) • Insufficient details (e.g., journal front or back covers)

Given the large number of papers meeting the refined inclusion criteria (*n* = 63), a sampling strategy was devised based on “thick description” (65, 66). Here, “thick descriptions” are loosely defined as those that offer sufficient detail to contribute to meaningful interpretations of the literature and, employing a common strategy, “thick descriptions” were contrasted against “thin descriptions” as relative measures of the level detail (67). All sources meeting the inclusion criteria were exported from Covidence (64) to Microsoft Excel (63), and assessed for richness using a 2 × 2 matrix based on the detail provided about the LHS and engagement activities. This sampling strategy allowed for the examination of contributor roles in different settings, contrasting different approaches to engagement. RG performed screening and sampling, with a small number of sources additionally screened and assessed for richness by TF and JW.

### 2.3 Analysis and interpretation

A data extraction template was developed in Covidence (64) and trialled on five sources. Following the hermeneutic method, data extraction was an iterative, multi-step process. For each source, the annotated copy from the full-text screening was printed for “close reading,” and the data extraction form was completed in Covidence (64). Emerging findings were added to post-it notes on a wall to organize, cluster, and capture relationships between observations. After this process was complete for each individual source, extracted data for all included sources were exported from Covidence (64) into Microsoft Excel (63) for aggregate analysis and comparison across sources.

Specific examples of engagement activities were coded inductively for each source. Multi-faceted activities were double-coded, for example a Patient and Family Advisory Council that contributed to recruitment was coded as both an advisory council and recruitment (68). During aggregate analysis, activities were coded progressively within and across sources. Similar activities were then consolidated to create a taxonomy of engagement. As a crude measure of frequency, sources were counted once for each type of engagement activity that they described. After inductive coding, the International Association for Public Participation (IAP2) Spectrum of Public Participation (69), herein IAP2 Spectrum, was used to deductively code individual engagement activities. The IAP2 Spectrum runs a gamut from “Inform” to “Empower,” with each successive position signalling contributors' increasing influence over decision-making (69). This framework was recommended by the SAG for analysing the nature of the engagement. Sources were counted once for each type of engagement activity that coded to a specific position on the IAP2 Spectrum (69). During aggregate analysis, coding was compared across sources for consistency and to determine the position of similar activities on the IAP2 Spectrum (69). Activities with insufficient detail to reasonably place on the IAP2 Spectrum (69) were coded as unclear.

A virtual “member reflections” exercise was performed in April 2023 to allow for collaborative input into coding engagement activities (66). Initial findings were presented to two SAG members (DR, SD), who then independently sorted 20 examples of engagement activities to a position on the IAP2 Spectrum (69) using the Optimal Workshop online closed card sort (70). Following a discussion about coding differences, RG reviewed the coding of all engagement activities. Finally, RG synthesized roles based on the coded descriptions of engagement activities, and notes and observations on engagement practices captured throughout the analysis.

## 3 Results

**Figure 1:**
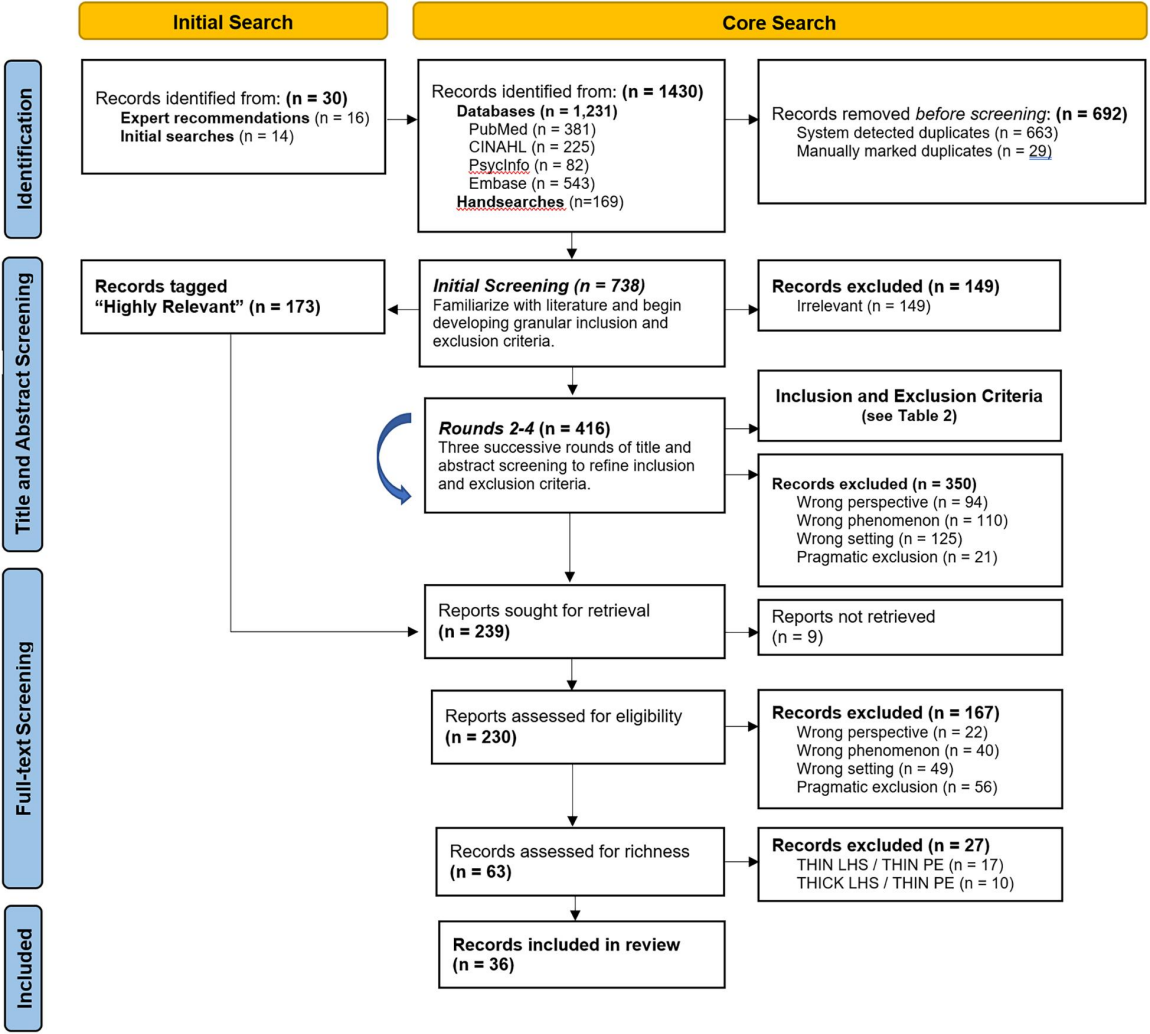
Summary of screening, sampling and selection. Adapted PRISMA diagram (71).

Of the 1,430 records identified, 63 sources met the inclusion criteria. After assessing for richness, 36 sources providing “thick descriptions” of engagement were included. The Preferred Reporting Items for Systematic Reviews and Meta-Analyses (PRISMA) flow diagram (71) was adapted to summarize the screening, sampling, and selection process (Figure 1). Findings characterize the literature and report on who is being engaged, where, when and how.

### 3.1 Study selection and characteristics

#### 3.1.1 Limited focus on engagement

The literature was highly heterogenous (Table 3), with only a third of sources (*n* = 12) focused primarily on reporting engagement activities. In addition to providing rich detail, these sources surfaced tensions around representation (68), between individual experiences and population-level data (72, 73), trust and different ways of knowing (15, 74, 75), and also documented barriers and facilitators to engagement (15, 68, 72, 73, 75–78). Yet, half these sources offered scant contextual information on the LHSs (15, 73, 75–77, 79). This highlights the value—and relative scarcity—of “thick descriptions” of engagement within the LHS literature (even when selected for), and the separation of rich detail on engagement from contextual information about the LHSs where these activities take place. Conversely, most sources describe a particular LHS, with varying levels of detail about engagement reported as a part of a broader program of work (*n* = 20). A small number of sources tackling aspects of LHS design (*n* = 4) also met the inclusion criteria by including examples of engagement in patient care settings.

**Table 3:** Summary of included studies by focus and type.

Source focus (# sources)	Focus	Source type	Examples
Engagement (*n* = 12)	Reporting primarily on engagement activities and/or engagement-related methods within a LHS.	Experiential Reports (68, 72, 74, 75, 78, 79)	• Contributors’ involvement in developing patient-led toolkits (68) and Patient-Reported Outcome Measures (PROMs) (72), creating and sharing resources (74) • Experiences establishing and maintaining a Community Advisory Committee (75) • Examining the role of engagement in system-level change (78) and over time across an LHS (79)
		Qualitative Studies (73, 76, 101)	• Interviews with patients and employees about patients’ roles in LHS governance (76) • Interviews with researchers and focus groups with patients about role for patients in evidence synthesis within LHSs (73) • Interviews with researchers conducting community-engaged research (CEnR) in an LHS (101)
		Participatory co-design methods (15)	• Participatory codesign workshops to explore ways to engage contributors in data-driven LHSs (15)
		Conceptual paper with examples (77)	• Explores the role of co-production within LHSs with an emphasis on role of contributors (77)
		Scoping review with examples (105)	• Literature review of coproduction in LHSs (105)
Specific LHS (*n* = 20)	Reporting on the planning, implementation, and/or evolution of a LHS. Engagement is a component of broader activities within a LHS.	Experiential Reports (80, 82–92)	• Evolution of LHS approaches in clinical settings and/or from previous research and/or quality improvement functions in health systems (80, 85, 86, 88, 93) • Establishing new LHS infrastructure, e.g., integrating data across multiple LHSs (89) and supporting pragmatic clinical trials (82) • Experiences of networked approaches, whether establishing new LHSs (87, 90–92) or operating large networks (83) • Describe LHS approach to designing clinical services (84)
		Qualitative Studies (104)	• Interviews with staff about experiences on implementing an LHS (104)
		Mixed Methods Studies (18, 96, 102)	• Using LHS to examine COVID-19 response in a clinical setting (18) • Identifying and prioritizing measures for tracking LHS performance (96) • Multistakeholder participatory approaches to exploring knowledge translation within specific LHSs (102)
		Protocols (97, 98, 103)	• Literature review and multiple case study to examine health system learning with Indigenous communities in Northwest Territories (97) • Mixed methods study, co-designed with patients and staff, to optimise long COVID care in the UK (98) • Establishing early intervention services for psychosis in Québec (103)
LHS design (*n* = 4)	Addressing one or more aspects of general LHS design illustrated with examples.	Experiential Reports (81, 94, 95)	• Explores the value proposition for LHSs from perspectives of clinicians and patients (81) • Organizational infrastructure (structures and processes) to support LHSs (94) • Practices for pursuing health equity within LHSs (95)
		Conceptual papers with examples (99)	• Proposes a “value-creation architecture” to mobilize and integrate the resources of health system actors in continuous improvement (99)

#### 3.1.2 Largely narrative reporting with limited co-authorship by contributors

Most papers (*n* = 22) took the form of an experiential report that describes the firsthand experience of an LHS (68, 72, 74, 75, 78–95). Sometimes experiential reports were published explicitly as “use cases” (95), “real-world examples” (81), case studies (68, 92) or commentary (80), but often without providing specific methods [e.g., (78, 79, 92)]. Notably, only nine sources explicitly reported contributors as co-authors in-text and/or in the author information (15, 76, 77, 79, 80, 87, 96–98). While this suggests limited co-authorship by contributors, this may be an underestimate. For example, two sources used group authorship—CERTAIN Collaborative (72), “the pSCANNER team” (89)—where a full list of people involved was included at the end of the paper (whether those listed included contributors was unclear). Contributors may also identify based on professional roles within health systems but also have lived experience [e.g., (77)]. Still, this suggests that contributors may be infrequently involved in reporting engagement practices within LHSs.

#### 3.1.3 Young and fragmented

All included sources were published in 2013 or later, with most sources (*n* = 31) published in 2017 or later. Nearly a third of sources (*n* = 10) were published in 2022, the year that the searches were performed. While the recency of the literature may improve its relevance to the current policy window, the sequential delays between the coining of LHS in the 2007 National Academy of Medicine (NAM) report (6) (the definition most cited amongst the sampled literature), the development of LHS models with an active role for patients, and the reporting of patient engagement activities may signal a tendency for engagement to be added in after implementation, developing over time as LHSs mature (72, 79, 82). Additionally, much of the literature is fragmented between journals with different topic areas, with 36 sources published in 26 journals. Only one source was published in a journal focused specifically on engagement (*Health Expectations*)—suggesting that the academic literature describing engagement activities within LHSs is relatively isolated from the broader peer-reviewed literature on engagement.

#### 3.1.4 Variable use of theory

Theory is used in the literature to situate engagement within LHSs; guide engagement activities; organize collaboration; implement and scale interventions; and critically examine social processes (Table 4). Despite the existence of numerous patient engagement frameworks (48), engagement functions were generally described as a component of broader LHS models. Only two sources used engagement frameworks to guide activities (72, 76), with a further two sources highlighting engagement frameworks that helped orient publicly-funded health systems towards engagement over time, namely the Strategy for Patient-Oriented Research (SPOR) in Saskatchewan, Canada (80) and the Person-Centred Practice Framework in Sweden (78).

**Table 4:** Theoretical traditions used in the sampled literature.

Theoretical perspectives	Use in literature	Examples
LHS models	Situate engagement within broader LHS model. The relevant theoretical construct is listed in parentheses.	• Levin et al. (84) describe the components of LHSs as aligning with Menear et al.'s (4) LHS framework (‘Social’) • Nash et al. (85) organize efforts around the nine criteria in Psek et al.'s (136) LHS framework (‘People and Partnerships’) • Davis et al. (86) report based on the four domains of the NAM LHS model (137) (‘Patient-Clinician Partnerships’) • Cassidy et al. (18) use the Lavis et al.'s (5) core LHS characteristics as a framework to study an emerging LHS (‘Engaged Patients’)
Engagement frameworks	Guiding specific engagement activities	• Grob et al. (76) adapt Carman et al.'s (138) framework for patient and family engagement to create a framework for engagement in LHS governance • Devine et al. (72) use Mullins’ 10-step process for patient engagement retrospectively
	Formative impact on system-level approach to engagement	• Strategy for Patient-Oriented Research (SPOR) in Saskatchewan (Canadian province) (80) • Person-Centred Practice Framework in Sweden (78)
Organizational theory	Approaches to collaboration including aligning goals, infrastructure (policies and procedures), communications	• Several sources draw on Fjeldstad et al.'s (100) Actor-Oriented Architecture (88, 90, 99) • Squires et al. (91) use relational coordination theory to guide collaborative tasks • Myers et al. (93) use constructs from the Collective Impact Model and the Interactive Systems Framework for Dissemination and Implementation to create organizational infrastructure to support people implementing interventions together
Implementation theories and frameworks	Structured processes for implementing interventions and scaling up changes	• Consolidated Framework for Implementation Research (CFIR) (93, 102) • Expert Recommendations on Implementing Change (ERIC) (102) • Template for Intervention Description and Replication (TIDieR) framework (104) • Plan Do Study Act (PDSA) cycles (87, 92, 93) • Keck et al. (74) invoke Metcalfe's Law to argue that the “value of a service to a user depends on the number of other users” (p. 2)
Critical social theory	Challenge social systems including ways of knowing	• Milligan et al. (97) plan to use two-eyed seeing or *Etuaptmumk,* an approach developed by Mi’kmaw Elder Albert Marshall that “values both Indigenous and western ways of thinking,” and “allows for reflexive consideration of the merits, limitations and challenges of different knowledge systems” (p. 4) • Knowles et al. (15) use ‘testimonial injustice’ (exclusion of people based on identity) and ‘hermeneutic injustice’ (exclusion or undervaluing of knowledge) as lenses for informing stakeholder discussions about which perspectives are excluded from patient-centred LHSs • Parsons et al. (95) describe the use of critical social theory for health inequities and addressing power-imbalances

More commonly, organizational theory is used to guide collaboration, and implementation science frameworks used to implement and scale interventions. Notably, there is a small cluster of sources with overlapping authorship that describe the use of “Actor-Oriented Architecture” (88, 90, 99), developed by Fjeldstad et al. (100), to theorize aspects of collaboration. Amongst these, Murray et al. (88) emphasize that organizations can be designed to support people to prioritize and solve problems—reflecting distributed leadership as a mechanism for collaborative priority setting. Overall, there is a notable lack of critical analysis of social processes. Milligan et al. (97) offer a strong critique, arguing that the “rather narrow orientation toward clinical research and health service delivery neither adequately captures the breadth and depth of relevant theory from other research traditions…nor promotes a model that prioritizes nonclinical or tacit forms of knowledge” (p. 2). There are, however, a handful of exceptions drawing on critical social theory to challenge inequities arising from colonialism, social marginalization, and/or established hierarchies of knowledge (15, 95, 97).

### 3.2 Findings about engagement practices

#### 3.2.1 Who is engaged in LHSs?

**Figure 2:**
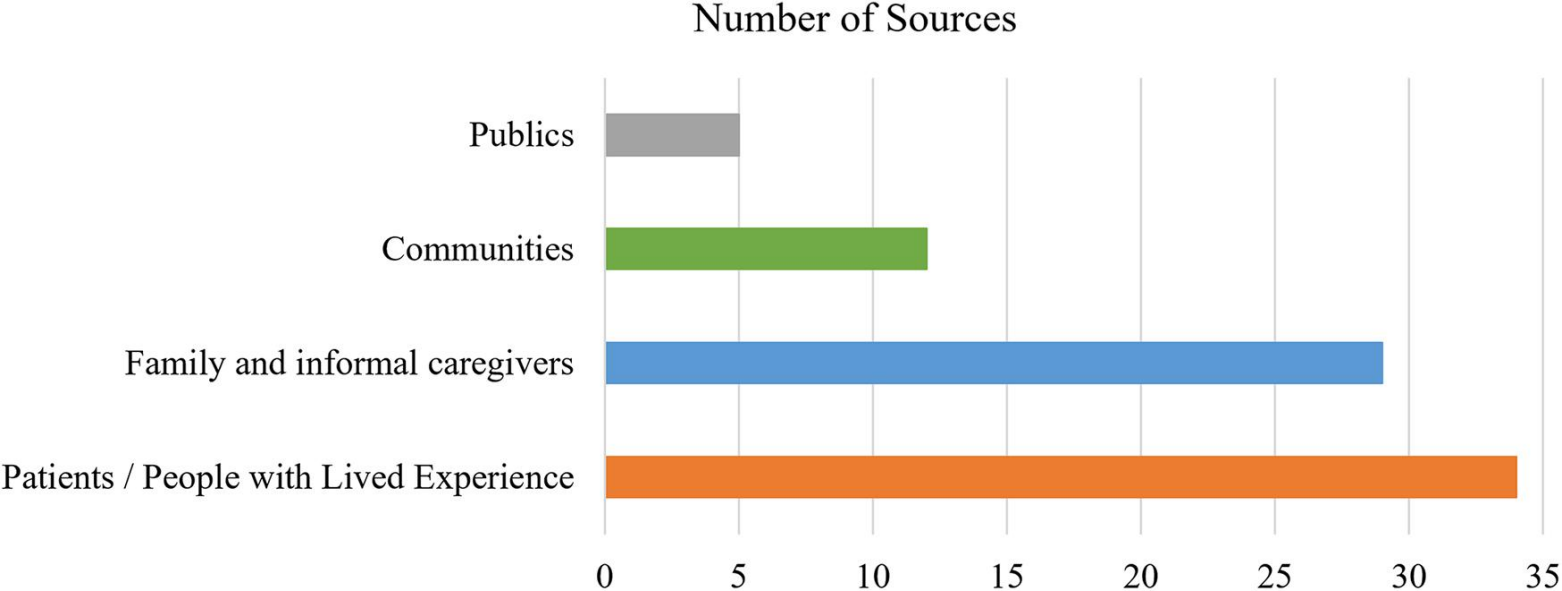
Number of sources engaging each contributor group. The total number of sources exceeds the number of included sources because nearly all sources engaged more than one group.

Most sources reported engaging more than one type of contributor (*n* = 30). Patients or people with lived experience of a health condition (*n* = 34) were engaged most often, followed closely by family and informal caregivers (*n* = 29) (Figure 2). Engaging contributors alongside other stakeholders was seen as advantageous, for example, in describing the design of the Collaborative Chronic Care Network (C3N), Seid et al. (90) noted that “taking a multi-stakeholder perspective forced the design team to consider the new system not as a system for doctors or a system for patients, but rather as a system for people” (p. 7)—an approach aligned with the quadruple aim central to the value proposition for many LHSs (4) and fitting pragmatic approach common to POR (21).

Engaging with communities was less common (*n* = 12) but also provided the only two instances where patients were not engaged. Community engagement focused on addressing persistent health inequities rooted beyond healthcare systems (97, 101). Notably, the term “community” was used variably, often making it difficult to determine who was being involved. Perhaps because the concept is nebulous and the practicalities of engaging large groups challenging (90), community perspectives were typically accessed via individuals: community leaders (83), organizers (90), representatives and/or members (75, 82, 85, 95, 101)—raising the thorny issue of representation (75). There were less frequent references to broader community outreach (75, 93) and partnering with community organizations (95), and these approaches were most common in LHSs with strong traditions of participatory community-based research (75, 101). Nonetheless, “community” tends to be treated as cogent “categories of shared identity” [(101), p. 2], often with scant reference to tensions within and between groups or the potential for intersecting identities.

Community engagement was also characterized by more a relational approach. For example, Seid et al. (90) frame LHS models as capable of appealing to solidarity, and thus supporting a shift from “health care as a transaction to shared work” (p.5). However, this focus on shared enterprise does not necessarily challenge the underlying assumptions about health or ways of knowing within these systems. Milligan et al. (97) emphasize how Indigenous knowledge systems, including a relational approach to well-being, can be at odds with colonial health systems that are typically rooted in Western traditions and derived from a biomedical model that privileges individual patient autonomy. This epistemic relationality presents a more fundamental challenge for LHSs, and the ways in which contributors' expertise and knowledge is valued within them. Notably, references to “publics” were rare (*n* = 5), amorphous, and often presumed, e.g., Levin et al. (84) indicated “patient partners provided a welcomed perspective on concerns and questions relevant for members of the patient community and the greater public” (p. 5). As a result, publics were not considered a distinct category for analysis.

Finally, examining who was being engaged provided insight into the potential nature of their contributions. There was considerable variation in the terms used to describe contributors, which were grouped to reflect their perceived expertise (Table 5). While the emphasis is clearly on lived experience of patients and caregivers, less common terms highlight potential shifts, for example a growing emphasis on cultural and/or organizational knowledge and relationships [e.g., Elders and Knowledge Keepers (97), veterans (102)]. Despite relatively few search terms emphasizing the relationship to the LHS, several were identified in the literature, e.g., actors (95, 100), coproducers (77), or “improvers” (74)—highlighting experience with the LHS as a sought-after form of expertise.

**Table 5:** Terms describing contributors, grouped by types of contribution.

Type of contribution	Terms used (# sources using term, if more than 1)	How knowledge and expertise are framed
Knowledge of health and/or supporting patients	Patients (*n* = 33) Family (*n* = 22) Caregiver (*n* = 6) Patient and/or Family Partner (*n* = 4) Patient and/Family Advisor (*n* = 4) Parents (*n* = 2) Patient Advocates (*n* = 2) Patient/caregivers Patient Leaders Patient representatives Patient and Parent Partners Patient/Family advocates Patient/Family Leaders Patient/Family representatives People living with cancer Representatives [patients/families] Self-Advocates	Firsthand experience of health-related condition and/or using a health system for seeking care for that condition, whether as a patient or caregiver—although sometimes those roles are treated as distinct.
Identifiable Groups	Communities (*n* = 12) Community members (*n* = 4) Community representative (*n* = 2) Veterans (*n* = 2) Community organizations Community organizers Community leaders Indigenous people Elders and Knowledge Holders	Perspectives engendered through belonging to an identifiable group. While broad terms are used often, the route to these perspectives individuals deemed to represent the group.
Awareness and Accountability	Public (*n* = 5) Naïve public Citizen	Broad categories describing whole populations serviced by the LHS, that should be aware of the LHS and/or which the LHS is accountable to.
Relationship to LHS	Actors (*n* = 2) Users (*n* = 2) Consumers Coproducers Clients End users Improvers Patient stakeholders Patient stakeholder informants Public contributors	The most instrumental of the categories, these individuals’ contributions are framed in relation to the LHS.

#### 3.2.2 When did engagement take place in LHSs?

**Figure 3:**
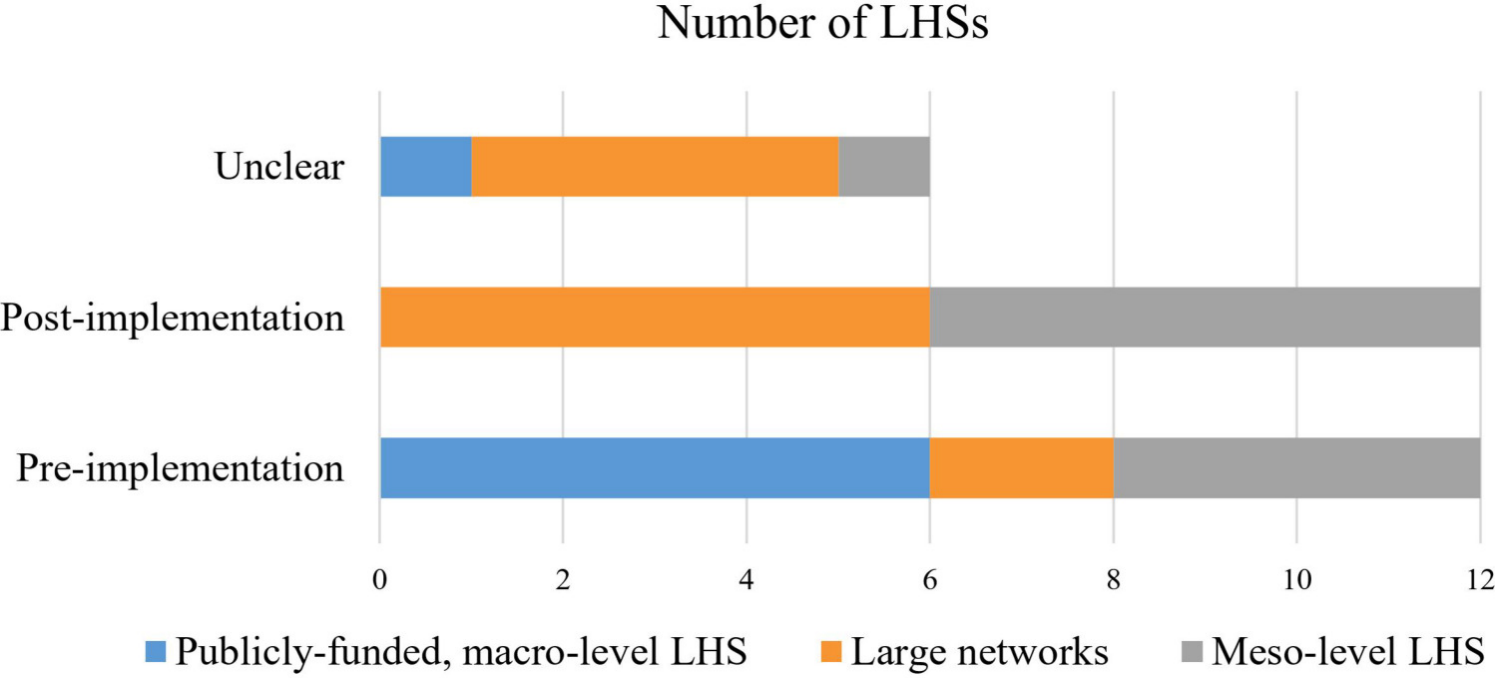
Timing of engagement relative to LHS implementation.

Timing of engagement was difficult to discern and largely inferred from the description of engagement activities. It was generally possible to determine whether engagement first occurred before or after the initial LHS implementation, with an even split between LHSs that engaged contributors from the outset or during early implementation versus those that engaged after implementation (Figure 3). This breakdown suggests that patients are often not default players, despite suggestions otherwise [e.g., (99)].

The exception was large publicly-funded LHSs, most of which initiated some form of engagement prior to LHS implementation (73, 80, 84, 89, 97, 98, 102, 103). The connection appears more tenuous at other levels of implementation, but there may be complex relationships at play. For example, all four meso-level LHSs that initiated engagement prior to implementation tended to be nested in larger organizational settings with established engagement traditions (77, 93, 96, 99, 104). This suggests that timing of engagement could be influenced by the presence of ongoing relationships and existing capacity for engagement. However, it is difficult to tell how much these findings are impacted by lack of reporting about the timing of engagement and/or when a health system starts using LHS terminology to describe itself.

#### 3.2.3 Where were contributors being engaged?

Contributors were engaged in 30 LHSs providing direct patient care (Supplementary Material S4). Most sources reported on LHSs operating in a single, high-income country: USA (*n* = 22), Canada (*n* = 6), United Kingdom (*n* = 3), or Sweden (*n* = 2). Exceptions included two sources offering examples from the US and Sweden (77, 99), and sources reporting on the Starzl Network for Excellence in Pediatric Transplantation, an international collaboration with partners in the US and Canada (87, 91), and ImproveCareNow (ICN), which has members in US, UK, Belgium, and Qatar (68, 74, 81, 90, 99, 105).

Clearly, engagement is happening in a variety of LHSs, operating in a range of care settings, serving diverse populations, and implemented at different levels, from smaller projects to large-scale networks. Half of the LHSs (*n* = 15) focused on specific diseases (68, 74, 81, 90, 99, 105) or services for specific disease (77, 99). Two LHSs focused on creating infrastructure: a learning network supporting discrete projects (83) and an LHS creating technical infrastructure to support data sharing between three large health networks (89). Additionally, some larger networks aimed to create unified standards of care across multiple organizations (87, 91). Nearly a third of LHSs (*n* = 7) were categorized as having more than one focus, for example Early Intervention Services for Psychosis in Québec is a disease-specific LHS implemented within a publicly-funded provincial health system (103).

Similarly, the boundaries between meso- and macro-levels of implementation are porous, for example some LHSs categorized as meso-level operate as single health systems but involve networks of facilities [e.g., (86, 94)]. Boundaries between LHSs are often grey. The Cincinnati Children's Hospital, an LHS early adopter in the US, acts as a common denominator for multiple LHSs, including operating internal services as LHS (Diabetes Centre), hosting larger networked LHS (ICN), and providing mentorship and support for LHS spin-offs (C3N). Nesting LHSs within a single health system was evident in other sources too, for example, the STRONG STAR Consortium (102) and Veteran Affairs Evidence Synthesis Program (73)—both positioned as LHSs, but taking different approaches to engagement within Veteran's Affairs (itself an LHS). These findings suggest that engagement is present in LHSs of all shapes and sizes, and points to opportunities to nest LHS approaches—providing learning opportunities within and between LHSs.

Unsurprisingly, given the variability of LHS implementation, several sources emphasized the need to tailor engagement to specific LHS settings (83, 94, 97, 101). Yet, there were no obvious connections between LHS focus or level of implementation and contributors' roles or the timing of engagement—with one notable exception. LHSs categorized as “publicly-funded, macro-level LHS” (*n* = 7) tended to focus on power-sharing and/or partnership as key motivators for engagement (73, 78, 80, 84, 89, 97, 98, 102, 103), as opposed to more pragmatic contributions to bounded projects and, as noted previously, all but one initiated engagement activities prior to LHS implementation (Figure 3). Analysing a diverse sample of LHSs helped to identify shared features, present across various settings, that differentiate LHSs from more siloed traditions of research and quality improvement (Table 6). These features impacted engagement—both positively and negatively—and thus represent important contextual considerations for designing patient-oriented LHSs and engagement practices within them.

**Table 6:** Common features of LHSs that impact engagement.

Feature	Advantages	Challenges
Dynamic systems	• Characterized by change, evolving over time (e.g., (84, 94); making engagement “perpetual work in progress” [(76), p. 7] • Responsive to feedback (15) • Flexible membership: people can join, leave and/or rejoin (68, 75, 102)	• Rapid change and concordant uncertainty contribute to a brittleness of engagement (90), with engagement dropping off or negatively impacted by the pace of change (18, 81, 82)
Nested approaches	• LHS implementation occurs iteratively at different levels, e.g., small projects within a single organization to large networks (see Supplementary Material S4) • “Starting small and learning deeply” [(95), p. 7] by creating opportunities to test and try different interventions • ‘Pockets of good’ can be identified and scaled up by networking smaller projects together (105)	• Risk of isolated “islands in a large system” [(78), p. 133] and friction, e.g., different values and priorities amongst stakeholders (81) • Engagement may become isolated from other functions (82) and/or underreported (68) • Lack of awareness about engagement activities and their impact (83)
Capacity and distributed leadership	• Longevity creates opportunities for establishing and engaging reservoirs of contributors (68, 79, 80, 99), and allows relationships to be maintained (101) • Ability to network within and across systems in order to create communities of practice (78, 81, 95) and ‘nimble networks’ (99) • Self-organization and distributed leadership (68, 88, 97, 105) • Contributors’ involvement in service design (77, 99)	• Commitment of funders crucial to sustainability and longevity (93)—reflected in publicly-funded LHS in Canada (80) and Sweden (78) • Power imbalances between contributors and other health system actors (90, 97, 99) • Difficult to establish culture of learning, and lack of shared goals and values (86)
Relationship with contributors within and beyond patient-care settings	• Can include patients and clinicians by default (99), with patients shifting from beneficiaries to members (74) • Connections beyond direct care settings, occasionally through data linkage and/or sharing (81, 84), but mostly through cross-sector partnerships, e.g., with schools (88, 95) and/or other community-based providers (79) • Contributors’ roles seen as core feature e.g.such that success is linked to “moving people up the ladder of engagement” [(74), p. 2] and engagement is seen as a sign of LHS maturity (82)	• No obvious or single path to deepening engagement practices within LHSs (82) • Engagement is valuable, but hard to measure and intangible suggesting an invisibility that complicates efforts to understand and evaluate impact (83) • Misunderstanding about roles (79); contributors unaccustomed to new roles (72) • Create new burdens on contributors (104) or cause burn out (101) • Inadequate representation (68, 73, 75, 76) • Underappreciation of community engagement methods (101)

#### 3.2.4 How were contributors engaged in LHSs?

Engagement activities (*n* = 192) were grouped into a taxonomy of engagement outlining seven functions (Table 7). All sources reported more than one type of activity. The most common engagement activities have strong traditions in patient-oriented research and/or quality improvement: advisory councils (*n* = 20); informal roles for individuals (*n* = 15); patient surveys (*n* = 16); training and education (*n* = 13); working groups (*n* = 13); creating resources (*n* = 9); providing testimonials (*n* = 7). These activities are consistent with the tendency for LHSs to build on internal research and quality improvement work [e.g., (75, 101)] or be designed *de novo* by partners drawing on existing traditions of engagement [e.g., (80, 90)]. Confirming the barrier noted by Menear et al. (4), priority setting activities were rare (*n* = 4). The lack of engagement in priority setting may be the product of delayed engagement, for example half of large networked LHSs reported on engagement activities occurring post-implementation (Figure 3), and is consistent with challenges noted in a CIHR SPOR evaluation (106). However, the relative rarity of these activities could also reflect a lack of reporting about timing of engagement and/or these functions may be assumed within references to other activities, e.g., governance committees.

**Table 7:** Taxonomy of engagement activities.

Engagement activity (# sources)	Description	Source(s)
Group work		
Advisory Councils or Groups or Committees (*n* = 20)	One or more defined group of contributors, e.g., patients, parents or caregivers, who act as advisory body to the LHS, providing advice across several functions and programs. Typically takes the form of a council, committee or group, although the composition, structure, and level of engagement varies.	(18, 68, 72–76, 79–81, 85, 86, 88, 89, 92–94, 98, 101, 102)
Governance Committees (*n* = 9)	Governance committees or structures with considerable control and oversight over program and/or LHS design. Multiple stakeholders, although composition varies.	(79, 81, 82, 84, 86, 88–90, 97)
Working groups (*n* = 12)	Typically brings together stakeholders, often with mixed roles, to work collaboratively on a specific subject or project over time. Often tied to programmes of work or workstreams and involved in implementation.	(68, 74, 75, 78, 79, 83, 84, 87, 88, 95, 96, 98)
Group working sessions (*n* = 10)	Range of activities, virtual or in-person, that bring people together for a specific function or interaction.	(76, 82, 88, 90–92, 94, 95, 97, 103)
Individual roles		
Compensation unclear (*n* = 14)	Roles for individual patients within LHSs, where the nature of the role and/or compensation are unclear.	(18, 72, 73, 75, 76, 79, 80, 88, 89, 92–95, 102)
Paid or funded (*n* = 4)	Funded and/or paid positions for individual patients within LHSs.	(75, 86, 99, 102)
Training and education		
Training and education programs (*n* = 12)	Capacity building activities and resources ranging from formal programs and platforms to passive distribution of education materials. Patients’ roles range from co-designers and co-educators to trainees.	(68, 74, 77, 78, 80, 82–84, 88, 93, 94, 103)
Mentoring (formal or informal) (*n* = 3)	Explicit references to mentorship at different scales from continuous one-to-one support to group settings to pairing programs for system-level mentorships.	(74, 79, 103)
Specific contributions		
Patient surveys and experience tools (*n* = 13)	Surveys and other online tools gathering data on patient experience. Typically takes the form of surveys or questionnaires, often captures patient-reported data such as Patient-Reported Outcome Measures. Can include methods to share aggregate and/or patient-level data, such as data dashboards.	(18, 72, 74, 76, 78, 83–86, 96, 103–105)
Priority setting activities (*n* = 4)	Activities designed to allow stakeholders to jointly set project and/or system priorities.	(73, 90, 91, 99)
Testimonials (*n* = 7)	Sharing patient experience through narrative, stories, and case studies. Often through presentations in group settings or over digital channels.	(73, 75, 76, 78, 86, 90, 91)
Creating resources for patients, caregivers, communities, and publics (*n* = 9)	Creating resources for patient and public audiences, in various formats including leaflets and toolkits. Emphasis on plain language and information relevant to patient and/or caregiver experience. All examples had a clear role for people being involved, ranging from patient-led efforts at co-production with multiple stakeholders to inviting patients and caregivers to review and provide feedback.	(68, 73, 74, 79, 81, 84, 99, 102, 105)
Advocacy (*n* = 5)	Activities and/or resources explicitly badged as patient advocacy.	(68, 76, 87, 91, 102)
Communications		
Website, online platforms and portals (*n* = 9)	Online spaces for sharing information and collaborating, includes websites and blogs, online project management tools and interactive patient portals. Spaces typically public but can be private.	(18, 68, 72–74, 84, 87, 99, 105)
Direct communications (*n* = 5)	Regular direct communications via any channel to maintain connections and share information. Typically, conference calls and/or email.	(68, 74, 87, 92, 99)
Regular meetings and gatherings (*n* = 4)	Regular meetings and gatherings, virtual or in-person, described primarily as touchpoints with stakeholders and opportunities to share progress.	(87, 95, 98, 99)
Social media (*n* = 5)	Patient-related social media activities, including ‘private’ spaces created on public platforms.	(18, 68, 73, 90, 92)
Co-production and participatory methods		
Service co-design and co-production (*n* = 9)	General references to contributors working together with other stakeholders to the design of clinical services, facilities, or resources. Invoked to communicate a substantive role for contributors including patient-led activities. Includes references to co-development and co-design.	(76, 84, 88, 92, 95–98, 104)
Personas (*n* = 2)	Constructed narratives, typically describing archetypal service users. May be based on experiential knowledge and/or composite of first-person experiences.	(15, 90)
Stakeholder mapping (*n* = 1)	Process of creating a matrix of stakeholders (individuals, groups, organizations) who have interest in a particular LHS, program/project, service, activity and/or resources.	(15)
Mind mapping and dot voting (*n* = 1)	"Paper-based free ideation around the study aim, and individual and group ranking of suggestions.” [(15), p. 105]	(15)
Speculative modelling (*n* = 1)	Stakeholders co-develop worst (“Dark”) and best (“Utopian”) case scenarios. This exercise uses “design provocations” to pose challenging questions and soft system modelling to qualitatively produce models of working."[(15), p. 105]	(15)
Affinity mapping (*n* = 1)	"Thematic analysis of discussions, presented visually using whiteboards and post-it notes, to organize and collate emerging understandings, capture and compare different points of view and agree on key findings."[(15), p. 105]	(15)
Tabletop modelling (*n* = 1)	"Mapping activity with mini-figures chosen to represent key stakeholders, to focus on the specific processes of interaction required to realize a goal in practice."[(15), p. 105]	(15)
CareMaps (*n* = 1)	A specific method using “visual tools diagramming a patient**'**s support systems providing insight into the patient**'**s “ecosystem” of care.” [(95), p. 4]	(95)
Supporting engagement		
Patient recruitment (*n* = 8)	Activities and resources designed to support recruitment of patients, includes patient registries, policies, and recruitment materials.	(68, 75, 76, 79–81, 89, 95)
Peer support and organizational structures (*n* = 7)	Creation of organizational structures (e.g., peer networks, engagement offices), and resources, strategies and/or policies to support engagement within LHSs. Typically created and/or maintained by LHSs but relate to peer support functions.	(76, 78–80, 98, 102, 105)
Funding and awards programs (*n* = 1)	Funding and/or awards that provide compensation for patients and/or support patient-led research.	(75)
Pre-meeting surveys (*n* = 1)	Surveys distributed to patients prior to multi-stakeholder meetings to inform meeting agenda and content for breakout sessions with patients and family.	(91)

The taxonomy of engagement provides practical examples of how contributors are being engaged in LHS, but also highlights shifts in engagement methods, particularly towards co-production (15, 76, 84, 88, 92, 95–98, 104) and, to a lesser extent, participatory methods (15, 95) that support community engagement. Existing approaches are evolving, e.g., the growing use of social media (18, 68, 73, 90, 107) and a shift from deficit-based training towards mentorship (74, 79, 103). However, newer approaches are less common, and the lines between old and new ways of engaging are often unclear. For example, in some instances, social media was used to involve patients in dialogue, e.g., the creation of an online community with “more than 150 people posting more than 1,700 messages” in a “private social media site” [(90), p. 5]. Conversely, social media channels were more commonly used to disseminate information [e.g., (18, 73)].

**Figure 4:**
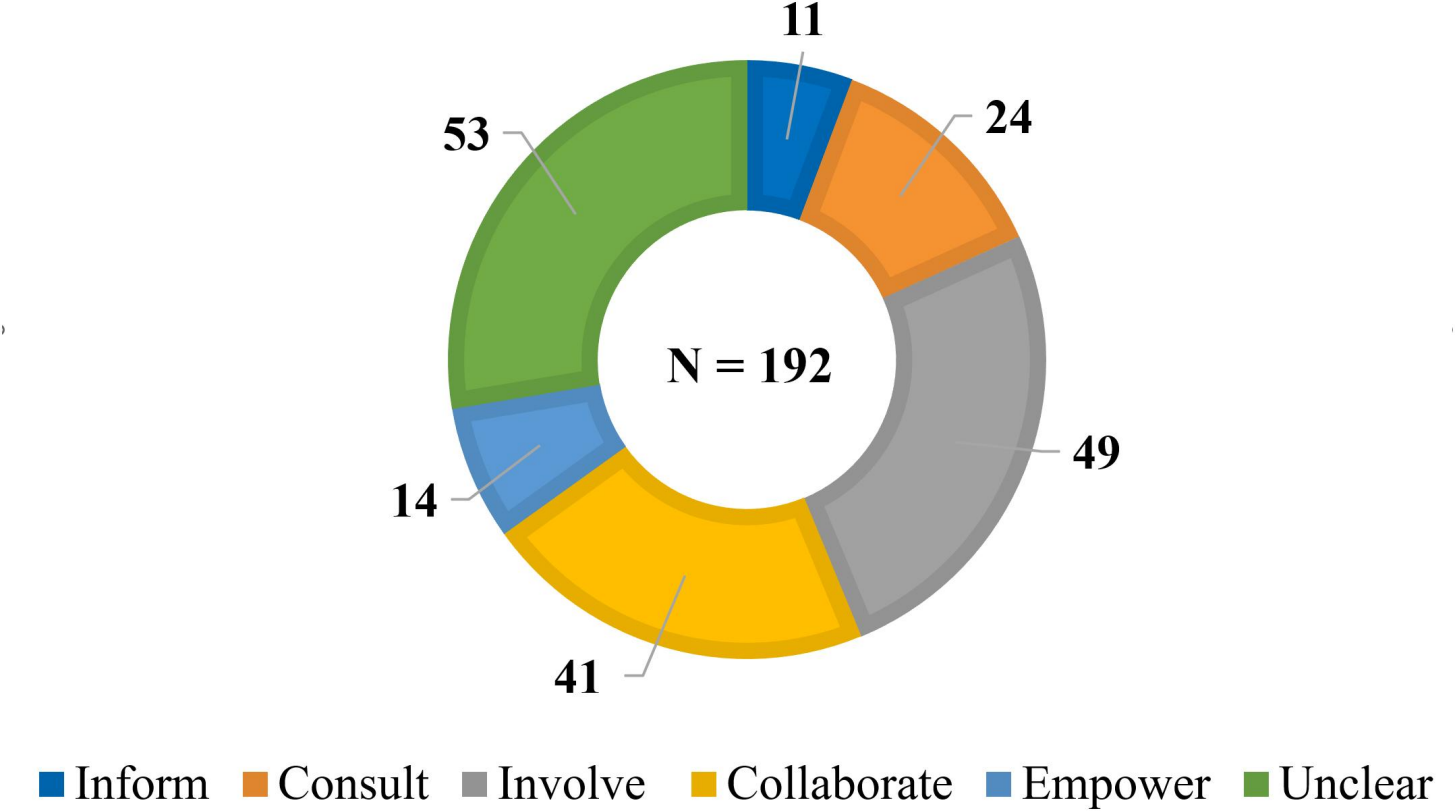
Number of engagement activities at each position on the IAP2 Spectrum.

Nearly two-thirds of the 192 engagement activities were mapped to a position on the IAP2 Spectrum (*n* = 139) (Figure 4). The aggregate data suggests contributors' influence over decision-making remains limited in LHS settings, with only a small number of activities coded to “Empower” (*n* = 14). Roughly half coded along the low- and middle-spectrum: “Inform” (*n* = 11), “Consult” (*n* = 24) and “Involve” (*n* = 49). While the descriptions of these activities suggest that their influence over decision-making is relatively bounded, SAG members noted that these activities can still serve important functions and be appropriate, if aligned with engagement goals. More than a quarter of engagement activities were coded unclear (*n* = 53)—highlighting the need for more descriptive and evaluative reporting of engagement practices.

**Figure 5:**
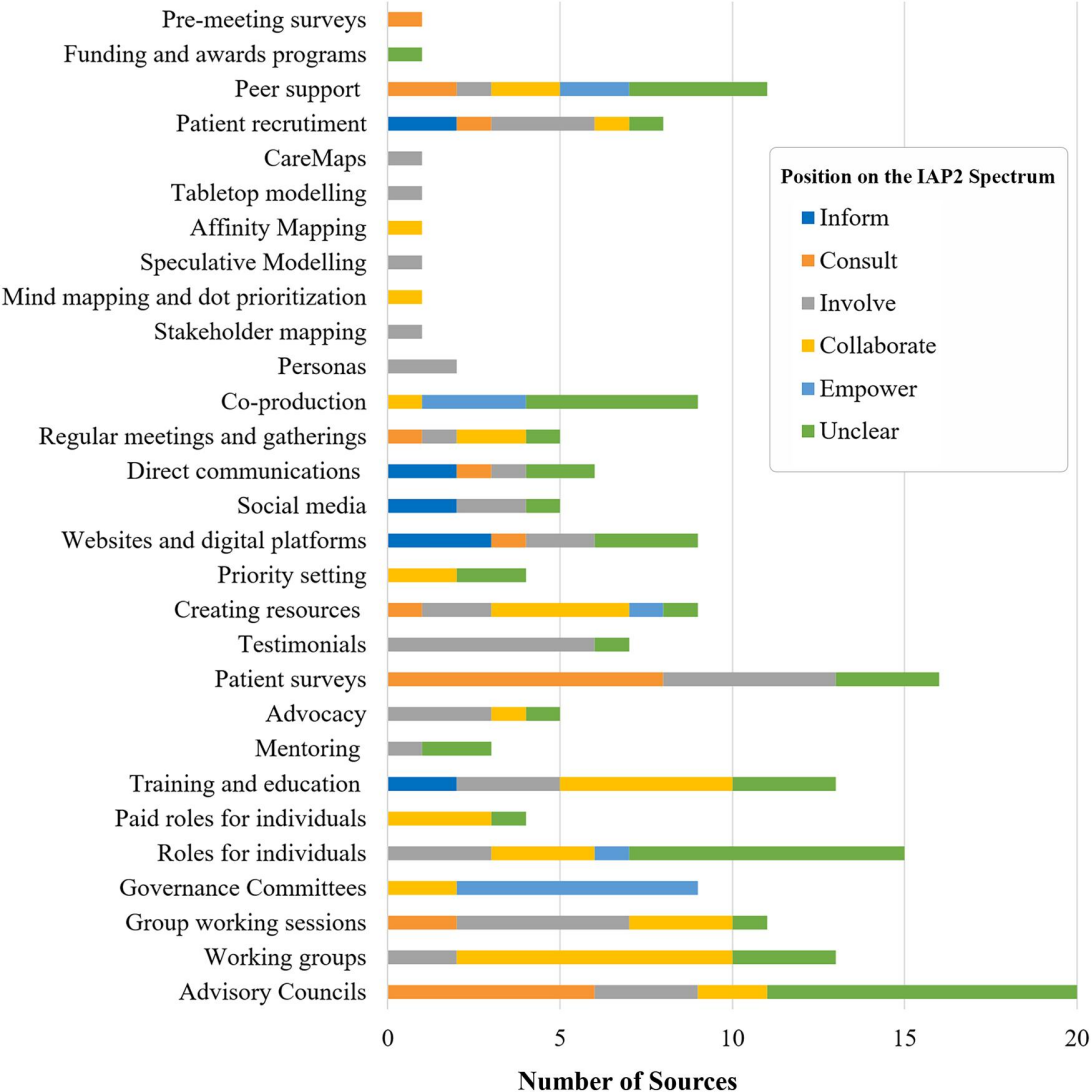
Engagement activities coded by position on the IAP2 Spectrum.

The breakdown of IAP2 Spectrum coding by activity provides a more nuanced view. Clearly design matters, with the same activity often occupying different places on the IAP2 Spectrum (Figure 5). This variation usually reflects differences between LHSs but, on rare occasions, the same type of activity occupied different places on the IAP2 Spectrum within the same LHS. These differences suggest that the nature of engagement cannot be taken for granted. For example, while co-production is billed as fundamentally collaborative, it was often invoked without details about the methods involved. As a result, about half of these general references were coded as unclear (84, 88, 92, 96, 104). Conversely, some references to co-production impacted decision-making sufficiently to be coded “Empower” (76, 95, 97), but the participatory methods associated with these approaches were coded as either “Involve” or “Collaborate”—suggesting co-production can also be more than the sum of its methodological parts.

The influence of the contextual features that impact engagement in LHSs (Table 6) is also apparent in the ways contributors are involved. For example, while governance committees often clearly described their decision-making authority (nearly all coded “Empower”), contributors may be ascribed influence over decision-making by virtue of their membership regardless of how much influence they exert. Similarly, influence over decision-making was far less clear for advisory groups than for working groups. Yet, decisions (albeit not usually at the system-level) tended to be made and implemented directly by working groups, perhaps an illustration of the assertion that distributed leadership can provide a path to meaningful engagement within LHSs [e.g., (75, 87, 95)].

The transition from informal to formal roles for individual contributors also provided opportunities for distributed leadership. Informal roles are more common (*n* = 15), but often had vague responsibilities and little or no reference to compensation. The scope of work for these roles tends to be more loosely defined (if at all) and/or their relationship to the broader organizations less clear. In contrast, four sources describe paid or funded formal roles for contributors (75, 86, 99, 102) that had clearer scopes (only one was coded unclear). Formal roles tended to move beyond narrative work and advisory functions to exert control over research, engagement, and/or knowledge translation activities, for example “Patient Supporters” in the Self-Dialysis Unit at Ryhov Hospital conceived the unit, co-designed the service, and trained patients to perform self-dialysis (99). Formal roles can also allow contributors to shape LHSs without assuming leadership positions, for example as “Community Research Associates” who combine research skills with community knowledge to perform a narrow range of tasks (75). These examples illustrate how contributors can be embedded within LHSs and guide the daily work of research and health system improvement.

Additionally, the active involvement of contributors in data-related functions appeared to be both constrained and evolving. Half of attempts to reflect contributor perspectives were consultative (*n* = 8) (18, 72, 74, 84–86, 96, 103), and accomplished primarily through patient/client surveys and standardized data collection tools (e.g., Patient-Reported Outcome Measures or, less frequently, Patient-Reported Experience Measures). There is a tension between bespoke or open measures that capture personal experience and standardized measures, which can be more cleanly collected, easily compared, and used to generate population-level data (72, 98). Knowles et al. (15) argue the need “to expand big data of informatics with the rich data of narrative and experience” (p. 112)—a position echoed in the call for data that is meaningful to both clinicians and patients (104) and capable of empowering people experiencing inequities (95). This process could involve engagement focused not only on what data is collected and how it is interpreted, but also by whom. There are indications that this transition is happening within LHSs through formal roles for contributors, for example Myers et al. (93) describe peer researchers gathering and interpreting data. There were also several engagement activities that actively involved contributors in data-related functions by helping to select and/or co-develop symptom questionnaires and survey tools (72, 83, 84), or through usability testing (103) and co-developing designing data dashboards (104).

Notably, despite excluding advocacy-related terms from the search syntax, advocacy activities were described in a small number of sources. For example, a petition jointly created by physicians and parents “to support prioritizing children for pediatric donor livers” received more than 15,000 signatures, demonstrating the “power of a unified voice” to “facilitate collection action” [(87), p. 423].

#### 3.2.5 What role do contributors play in LHSs?

**Figure 6:**
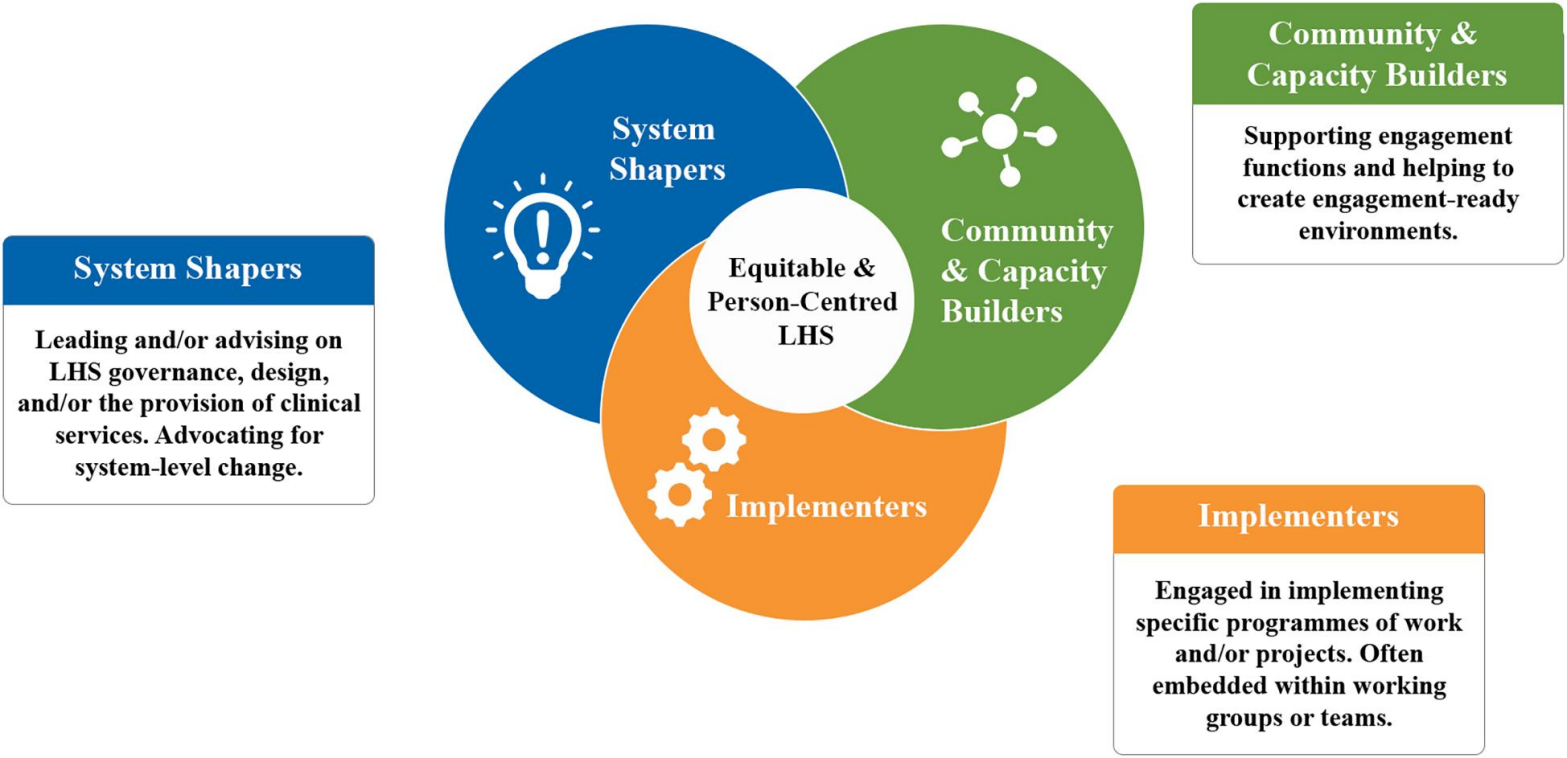
Functions of contributors in equitable and person-centred LHSs.

Ten distinct roles for contributors were synthesized, serving three broad functions within LHSs (Table 8). Contributors acted as System Shapers (designing and defining LHSs), Community and Capacity Builders (expanding and supporting LHSs), and Implementers (undertaking hands-on tasks) (Figure 6). Roles were flexible, varied, and often overlap with individuals sometimes performing several functions, along with the ability to come and go from roles. Three LHSs are associated with a single role for contributors (81, 85, 104); all other LHSs were associated with multiple roles.

**Table 8:** Roles for patients, caregivers, and communities within LHSs.

Role (# LHSs)	Description	Sources
System Shapers		
Leaders (*n* = 15)	Leaders operate at different levels, providing system-level leadership through multi-stakeholder committee involvement (e.g., membership on Steering Committees) and/or by leading or co-leading specific projects, work packages and/or activities.	(68, 73–76, 79, 81–84, 86–90, 92, 95, 97, 98)
Advisors (*n* = 13)	Providing contributor input into LHSs and/or project design, through dedicated advisory councils and/or committees (as distinct from organizational and/or LHS-wide governance mechanisms).	(18, 68, 72–76, 79–81, 86–88, 92–94, 98, 101, 102)
Service Designers (*n* = 6)	Contributing to the development of clinical services often through co-design.	(76, 79, 84, 92, 98, 100, 103)
Advocates (*n* = 3)	Petitioning for policy changes and/or other patient advocacy activities.	(68, 87, 102)
Community and Capacity Builders		
Peer Supporters (*n* = 10)	Providing support and/or mentorship, ranging from ‘one-to-one’ sessions to the development of peer-led resources or activities to support engagement.	(68, 74–76, 78–80, 87, 89, 99, 102, 103)
Trainers and/or Trainees (*n* = 11)	Involvement in design and/or delivery of training and capacity building activities and/or information resources.	(68, 74, 76–78, 80–84, 88, 92, 93, 103)
Reservoirs (*n* = 8)	Affiliation and/or membership with LHS, from formal and informal roles to joining patient registries to signing up for communications.	(15, 68, 74, 78–80, 90, 92, 95, 99, 105)
Implementers		
Storytellers (*n* = 8)	Sharing experience of health, care, and potentially engagement through various communications channels (e.g., blogs, presentations, testimonials).	(73–76, 78, 86, 88, 90, 97)
Consultants (*n* = 20)	Providing feedback through consultative processes, e.g., completing patient experience surveys or participating in rapid design sessions.	(18, 72, 74, 76, 78, 80–87, 90–92, 94, 96, 97, 99, 102–105)
Workers (*n* = 18)	Contributors are collaborators and/or team members involved in executing specific programmes of work and/or activities, often through specific projects and/or working groups.	(15, 68, 72, 73, 75, 76, 78, 79, 83, 84, 86, 87, 93–96, 98, 99, 102)

For LHSs described in more than one source, e.g., ImproveCareNow (ICN) (68, 74, 81, 90, 99, 105), it was common for different sources to describe different roles. Differences in contributor roles between sources could be due to the focus of the publication but, in at least some cases, sources indicated roles that changed over time. For example David et al. (68) describes self-directed changes to the ICN Patient Advisory Council structure and membership engagement, and how this adaptive process helped “meet identified community needs” (p. 8). Therefore, roles described for each LHS are unlikely to be exhaustive but rather provide a snapshot of engagement and, just like engagement activities, offer opportunities to tailor engagement practices to specific LHS settings. There is considerable flexibility for those designing LHSs, both in addressing system needs and matching contributors with roles that suit their interests and skills.

In line with the dearth of activities coded to “Empower” on the IAP2 Spectrum, the most common roles related to the pragmatic “Implementers” function and are associated with a narrower scope of engagement—either as “Consultants” (*n* = 20 LHSs) or “Workers” (*n* = 18 LHSs). Conversely, the roles associated with “System Shapers” and “Community and Capacity Builders” suggest intriguing possibilities to reimagine engagement to take advantage of the unique features of LHSs (Table 6). For example, within the “System Shapers” category, “Leaders” did not necessarily have influence over system-level decision-making but were able to effect change through distributed leadership. For example, formal roles such as “funded investigators” (86) represent a devolution of powers akin to what Hughes and Duffy (108) describe as “user-led research,” where people with lived experience direct work including defining projects, seeking funding and publishing results***.*** Defined to reflect distributed leadership, “Leaders” was the third most common role, present in nearly half of LHSs (*n* = 15 LHSs)—suggesting an expanding and possibly bottom-up influence for contributors over these dynamic health systems. Other examples include “Advocates” which reflect connections between LHSs and broader social context; “Service Designers” which reflects the embeddedness of contributors within clinical care settings; and “Reservoirs” which are well-suited to nested and networked approaches.

“Reservoirs,” where formal or informal affiliation connects contributors to LHSs, were also a key strategy for embedding contributors, and a way to balance between rapid change and potential longevity of LHSs (68, 74, 78–80, 90, 92, 95, 99, 105). Access to experienced contributors was seen as helpful for people new to engagement and a route to including more diverse perspectives (84). Reservoirs may also support flexibility, allowing contributors to take on and resign different roles over time. Practically speaking reservoirs were created via social processes (establishing and nurturing relationships), capacity development (training and mentoring), and organizational infrastructure, for example supportive policies, hiring engagement staff, formalizing contributor roles, routine communications (e.g., mailing lists), and patient registries. The impact of reservoirs depends on how they are used and maintained, and the ways in which contributors are called up. For example, patient registries can be passive pools occasionally dipped into (75, 78) or used to more proactively engage contributors, e.g., the Diabetes Centre Patient Registry, which is used to identify at-risk patients to involve and potentially support with an innovation fund (95). Having supportive organizational structures with engagement staff, e.g., dedicated office (76) or platform (80), is noted as a facilitator but warrants further exploration.

A closely related strategy involved embedding contributors as “Peer supporters,” whose involvement helped create engagement-ready environments. “Peer supporters” were involved in various peer functions, e.g., supporting engagement including through recruitment (75), onboarding (87), and helping other contributors to participate in or manage engagement activities and governance functions (79, 88, 89, 93). Peer functions were largely performed via groups, for example coalitions (80), collaboratives (76), networks (79, 98, 102). While mostly positioned as beneficial to the LHSs, contributors also expressed a desire for peer communities that provide forums to share experiences and support each others' engagement efforts (80). Peer functions were positioned near-universally as positive and supportive. For example, contributor involvement in recruitment is presented in a largely uncritical way with a focus on the benefits of attracting and supporting new contributors [e.g., (68, 75, 79, 80, 89)] and rarely problematized the potential bias for recruiting narrow perspectives. There was no discussion of what Vinson [(109), p. 3] calls “peer regulation”—a process of “orienting members’ action toward the stated values of a group”—and, by extension, the potential for “peer policing” or informal social control over contributors. More work is needed to understand how peers will relate to each other, as well as other health system actors, particularly those mediating peer functions such as engagement staff.

## 4 Discussion

This review provides an overview of engagement practices within existing and emerging LHSs. Unsurprisingly, given the flexibility of the LHS concept, the peer-reviewed literature on the topic is highly heterogenous and fragmented. In line with LHS models, engagement was frequently reported as a component of a larger programme of work, often limiting the detail provided. Despite these challenges, 192 engagement activities from 30 LHSs were examined to create a taxonomy of engagement (Table 7). Additionally, ten flexible and overlapping contributor roles were synthesized, which serve three core functions: “System Shapers” (designing and defining LHSs), “Community and Capacity Builders” (expanding and supporting LHSs), and “Implementers” (hands-on efforts). Taken together, these findings provide useful examples of how and why contributors have been engaged in LHSs, drawn from and applicable to a range of settings.

Critically examining engagement practices in LHSs demonstrates how engagement in these settings both builds on and is constrained by engagement traditions in research and quality improvement, which can be siloed (80) and vary in the extent of contributor involvement [e.g., (47, 48, 56)]. Having a culture of and capacity for engagement can be beneficial [e.g., (68, 72, 80, 86, 88)], but scaffolding to established practices risks perpetuating tokenism and other poor engagement practices (20, 106, 110, 111), as evidenced by the relative scarcity of activities coding to “Empower” on the IAP2 Spectrum (69). Few priority setting activities were identified and, when reported, the timing of engagement (after implementation in half of LHSs), suggests that contributors are not always included by default, despite these systems operating in direct patient care settings. These findings affirm and elaborate the documented “promise-practice” gap when it comes to engagement in LHSs (4, 16, 17).

There is also considerable latitude to define and refine engagement within the complex settings typical of LHSs. The nature of engagement activities is not inherent, with the same types of activities mapping to different positions on the IAP2 Spectrum (69)—variability that highlights the importance of how activities are structured and supported. Engagement activities are evolving, with the creation of formal roles, shifts towards mentorship over deficit-based training, and an emphasis on co-production including expanded roles for contributors in data-related functions. Contributors often play multiple roles within a single LHS, with new roles emerging that embed contributors within LHSs—particularly those associated with the “System Shapers” and “Community and Capacity Builders” functions.

In line with recent recommendations that contributors “be present and participate in” LHS design (112), embedded contributors become available to participate in deliberative system design, for example helping to network nested approaches; exercising distributed leadership; and nurturing relationships with other health system stakeholders.^3^ This relational approach makes them a part of “relational and self-organizing systems” [(113), p. 665], and aligns with a growing treatment of engagement as a landscape for mutual exploration (114), and complex, cooperative ecosystems mediated by dynamics between various actors (115). Embedding contributors could also strengthen and expand the small but distinct group of contributors recognized for their relationship to the LHSs and, by extension, knowledge of the health system. This shift would tap into a growing desire to capitalize on institutional knowledge held by experienced contributors (20), and increased calls for “systemic integration” of engagement to support LHS approaches [(116), p. 5] and for patient partner compensation (106, 116–118).

Yet, the relative scarcity of embedded approaches suggests that, while promising, embedding contributors within LHSs remains a largely untapped strategy. Considerable efforts have focused on embedding researchers in Canadian LHSs [e.g., (119)], but more work is needed to explore the practicalities of embedding contributors, for example organizational policies and compensation. There is also a need to evaluate contributors' desire for and experience of embedded functions, and to understand the perspectives of other health system actors, including engagement staff. This is particularly important given the potential of embedding contributors to exacerbate barriers to engagement, notably misunderstandings about contributor roles (79), challenges in adjusting to new roles (72), and creating burdens on contributors (104). When developing these new roles and ways of working, embedded contributors will need to navigate changing relationships with other health system actors, as well as with their peers. These interactions are shaped by the ways in which their knowledge and expertise are framed within LHSs (44, 120–122), values (113, 123), and by power dynamics (25). Although rarely invoked in the sampled LHS literature, social theory could help to explicate these complex social processes and provide useful lenses for further elaborating the social struggles that arise when embedding contributors.

Alongside the inwards shift to embed contributors, there is also an outwards expansion characterized by community engagement beyond direct patient care settings. In the sampled literature, community-based approaches often focus on addressing inequities and are distinct from patient and caregiver involvement. This aligns with calls for equity-centred engagement of distinct stakeholder groups to accelerate learning in LHSs (124), but this shift has important implications in the Canadian context. First, while the SPOR definition of engagement includes “affected communities” (Table 1), engagement has typically focused on individual patients and/or caregivers, with expertise derived from lived experience of health conditions and/or receiving health care. Recruitment largely relies on self-selection (125), with patients brought into stages of research, often to inform or consult on activities (106)—and not necessarily using participatory methods. Expanding engagement through existing networks may prove insufficient due to a lack of diversity amongst the existing patient partner community in Canada, which is skewed to older, white females (20). Furthermore, considerable effort will likely be needed to build relationships with equity-denied communities, and there were relatively few examples of LHSs engaging beyond the health system apart from a handful with cross-sectoral partnerships [e.g., (79, 86, 88, 95)].

By contrast, LHSs with a focus on equity tended to use participatory methods aimed at engaging and building relationships with communities, who bring experience beyond health and healthcare (75, 97, 101). This shift could be informed by the discourse about learning (121), and reflects “‘learning with’ community [which] entails authentic partnership, power-sharing and the co-production of knowledge” rather than more extractive and reductive approaches to “learning from” communities [(126), p. 2]. Additionally, the sampled literature suggests a need to engage communities in processes of *unlearning*. Beyond identifying and discontinuing practices that do not improve patient outcomes (82, 97), unlearning recognizes a need to “disassemble systems that have produced inequities, reimagine and create ones that produce equity, and overcome the inertia of the status quo” [(95), p. 4]. This involves examining not only patient outcomes but also changing “practices or policies that resulted in [the] current state” of the healthcare system [(95), p. 4]. This disassembly needs to address epistemic injustice or the undervaluing of some forms of knowledge (15)—a difficult task for health systems adherent to the hierarchy of knowledge associated with evidence-based medicine (127–129). The social processes underpinning learning and unlearning from communities will be important considerations for LHSs tackling inequities (93), particularly when engaging equity-denied communities where historic injustices compound current inequities (97), such that there is a need for “relationship repair” [(101), p. 7].

Therefore, the shift towards community engagement could bring the current pragmatic rationale for engagement into tension with more democratic rationales (25, 44), which enlist “broader social and ethical narratives around democratic representation, transparency, accountability, responsibility and the redressing of power imbalances” [as cited in (25, p. 6)]. As Dhamanaskar et al. (125) noted, the historical mixing of patient and public engagement obscures important theoretical differences in pragmatic patient-oriented approaches and more democratically motivated public engagement (124). This suggests that LHSs with strong POR traditions may need to rethink not just who is engaged but how and why, as well as what expertise contributors bring—particularly if aiming to improve health equity.

### 4.1 Strengths and limitations

Combining the hermeneutic method, with its focus on interpretive understanding, and the structure of the PerSPEcTiF framework was a key strength of this study. Coding and quantifying data added insight into the type, frequency, and nature of engagement activities, particularly through the application of the IAP2 Spectrum (69). This approach was flexible enough to include sources that supported critical reflections on how engagement differs in various LHS settings. However, to maintain a manageable scope, grey literature was excluded. This is an important limitation as strict word counts in peer-review publications may limit the level of detail provided about engagement practices and may bias the sample towards research, as opposed to other engagement activities that may not as routinely result in peer-reviewed publications, for example quality improvement initiatives. Additionally, screening, sampling, and analysis were also highly subjective; another reviewer may have selected a different sample, possibly arriving at different conclusions. The Stakeholder Advisory Group (SAG) helped to incorporate the perspectives of patients, engagement staff, and researchers, improving rigour and enriching the findings with alternative views (66), and increasing the relevance of findings (130).

Findings about individual engagement practices are also constrained by a tendency for easy measures of engagement (131) and “descriptive rather than evaluative” reporting (25, p. 2). This complicates efforts to understand the nature and scope of engagement activities, how they proceed over time, and their context (131, 132). Additionally, there is a relatively positive perspective on engagement that may arise from selecting for LHSs that value engagement enough to be doing it and/or publication bias. Where possible, sources describing engagement within the same LHS were selected to provide additional detail, and efforts were made to engage with critical themes. There are additional limitations arising from the literature itself. The variability in terms could lead to language-related blind spots. Publics were rarely engaged and/or essentially assumed via participation of patients, such that there was insufficient information to consider this group in the analysis. There was also insufficient detail to code the sociodemographic characteristics of contributors—information that is needed to better understand and support equitable engagement practices (106). Furthermore, the LHSs described in this sample operate in a small number of developed countries. This could impact on the applicability of findings in lower- and middle-income countries. Unique insights from these settings may also be missing, for example community-based health workers could provide useful models for formalizing roles and/or creating contributor reserves (133). Additionally, co-authorship by contributors was rare, which is out-of-step with increased calls for contributor acknowledgement (117, 118), and could materially impact how engagement practices are reported, as contributors experience engagement and view outcomes differently than other stakeholders [e.g., (106)].

Despite these limitations, narrative review “deals in *plausible* truth” [(40), p. 3]. This study offers an overview of engagement within emerging and existing LHSs—not an exhaustive account. If anything, this work reinforces that there is no complete sample or single way to do engagement in LHSs. It does, however, provide a snapshot of existing practices at the time of the search that highlights unique opportunities to design meaningful engagement within LHSs (Table 9), at a time when institutions are grappling with the realities of LHS implementation and SPOR is undergoing a refresh including a potential shift towards broader engagement and increasing community involvement (141).

**Table 9:** Summary of implications for LHS implementation.

Key messages
• Contributors in LHSs hold multiple, evolving, and overlapping roles in LHSs. Emerging roles suggest new opportunities for contributors to be involved in deliberative system design and to effect change through distributed leadership. The ten roles synthesized from the literature provide a starting point to design meaningful contributor roles in LHSs. • Engaging contributors, particularly communities, has an important role to play in designing LHSs as sites of both learning (e.g., gathering new data, generating evidence, or de-implementing low-value practices) and *unlearning* (e.g., problematizing existing practices, tackling epistemic injustices by valuing other forms of knowledge). • Embedding contributors in LHSs can support early and/or continual engagement, for example by creating formal positions, establishing reservoirs of contributors, and supporting peer functions. The resources required to support these strategies remains an open question, and more research is needed to understand organizational barriers and facilitators, as well as contributors’ perspectives on embedded roles. • Engagement in LHSs can be continually defined and refined to take advantage of the unique and dynamic context of the LHS, as well as common contextual features of LHS. For example, if taking a nested approach by engaging contributors in piloting an intervention within a broader LHS, consider how contributors might also support learning and/or awareness beyond the implementation setting and team. • The same engagement activity may have different goals under different conditions, and multiple engagement activities can be adapted and combined to engage contributors in the desired way. Engagement activities are also evolving in LHSs including an emphasis on co-production, the emergence of mentorship over deficit-based approaches, and greater involvement in data-related functions. • Community engagement is more common in LHSs tackling inequities and is fundamentally different from more pragmatic approaches to patient engagement. For LHSs with strong POR traditions, this may require new ways of working (e.g., more relational approaches, participatory methods, drawing on different theoretical traditions, and/or additional resources and organizational supports or policies). • Future research should expand and explicate contributor roles, paying particular attention to embedded opportunities and community engagement, as well as examining the impact of engagement activities on LHS outcomes and exploring negative and/or unintended consequences of engagement. Additionally, references to public engagement were rare and warrant more exploration.

### 4.2 Conclusions

Most LHS models call for an active role for patients and communities, yet contributor roles have arguably remained limited with few mechanisms for engagement in priority setting and system design. To help bridge this gap, this narrative review provides an overview of engagement practices in existing and emerging LHSs. Overall, engagement practices varied across the literature, with room to improve the role of contributors in LHSs. Emerging roles suggest new opportunities for contributors to participate in deliberative system design and challenge persistent health inequities. Future research should expand and explicate contributor roles, paying particular attention to embedded opportunities and community engagement, as well as examining the impact of engagement activities on LHS outcomes and exploring negative and/or unintended consequences of engagement.

## Footnotes

^1^Known as the Institute of Medicine until 2015.

^2^The term ‘stakeholders’ was used throughout the study, including with the Stakeholder Advisory Group, and was common in the sampled literature. Because of its colonial connotations, there have been efforts to identify alternative terms, for example ‘interest holders’ has been used elsewhere (26), and this language is likely to continue to evolve.

^3^This relational approach may be less well-suited to more positivist LHSs, for example where importance is pinned on randomization and reducing risk of bias (82) or the focus is on enabling consistent standards of care (91).
