# Supplementary material for: The role of patients, caregivers, and communities in Learning Health Systems: a narrative review
Source: Front Health Serv. 2025 Oct 21;5:1606124. doi: 10.3389/frhs.2025.1606124 (PMC12584131; doi:10.3389/frhs.2025.1606124)
Supplement: Supplementary file 1 [file Supplementaryfile1.docx]

Supplementary Material 1. Stakeholder engagement reported using the GRIPP2-SF

| Section and topic | Item | Reported on page # |
| --- | --- | --- |
| 1: Aim | To enlist specific perspectives into the research, avoid duplication of efforts, and connect findings to practice. | 6 |
| 2: Methods | A stakeholder engagement plan was drafted to seek support to recruit within Maritime SPOR SUPPORT Unit (MSSU) and Nova Scotia Health (NSH). Five stakeholders were purposively recruited to a Stakeholder Advisory Group (SAG). All SAG members (DR, MS, KH, SD, LM) completed a pre-engagement survey, and participated in 2.5 hour session to refine list of key terms and the research questions.  Following this meeting, all SAG members had the opportunity to review the draft search strategy for the core search cycle. One SAG member (MS) provided feedback on the draft search strategy. Preliminary findings were presented to two SAG members (DR, SD), who participated in a member reflections exercise, which involved coding a sample of engagement activities to a position on the IAP2 Spectrum (69).  SAG members also assisted with knowledge translation activities, including co-authoring this paper. SAG members also co-authored a poster presentation for Canadian Association of Health Services and Research (CAHSPR) Annual Meeting. A SAG member (DR) arranged and hosted a webinar sharing findings with the national Patient Advisory Network (PAN), as well as providing feedback on the presentation itself. SAG members also co-authored an abstract for the MSSU Maritime Health Research Summit, which was accepted and presented by the student lead (RG). | 6-7; 10; 19; 31 |
| 3: Study results | SAG members contributed to this research in several significant ways:   - Refining the research question to focus on examples of engagement in practice, and narrowing objectives (for example, removing evaluation from the scope as this is being investigated by others already); - Revising the list of key terms and key definitions (e.g. recommending not including advocacy-related terms), identifying several additional terms (indicated with ** in Table 1), helping to refine working definitions, and clarify parameters for the search; - Recommending the IAP2 Spectrum (69) as a framework for analysing engagement activities; - Providing feedback on the draft search strategy; - Highlighting some of the challenges and benefits of coding with IAP2 Spectrum (69), which led to the student lead (RG) doing another round of more conservative coding. - Providing critical feedback on the draft manuscript, thus shaping the discussion and highlighting important limitations.   Additionally, meetings and exchanges with the SAG members necessitated summarizing research, methods, and findings in accessible ways and at various stages of work – the minutes, slides, and information generated through these interactions became useful references. | 6-7; 10; 19; 29 |
| 4: Discussion and conclusions | Stakeholder engagement was crucial to the development of this project and influenced both its scope and findings in significant ways.  The positive impact that engagement had on this research may be related to several factors. SAG members were recruited from familiar organizations and were experienced contributors; most had existing relationships with the student lead (RG) and/or at least one other member of the SAG. SAG members brought considerable experience, from various perspectives, including deep knowledge of engagement practices within the local context. The mix of experience helped to contrast different perspectives on and approaches to engagement (particularly between patients and staff), enriching the work.  The student lead (RG) also had training and experience in patient-oriented research and sought to implement best practices, for example making administrative arrangements to pay SAG members who wished to receive an honorarium.  While time consuming to develop, the stakeholder engagement plan helped to ensure that engagement activities would meet degree requirements, clarify the purpose of engagement, and support recruitment.  Another important factor was early engagement. The SAG was involved from the outset of the project, and were thus able to shape the research from the questions through the analysis, despite a relatively limited engagement.  There were challenges too. Engagement added considerable effort for the student lead (RG), who had to invest time (engagement activities, but also planning and follow up) and money (honorariums were paid out-of-pocket). Another key challenge was the timeline. There was a long gap between the initial engagement and the member checking exercise, which may have contributed to a drop-off in interest amongst SAG members. This is partially due to the study design (e.g. no involvement in screening, sampling, and analysis) but also due to life events for both the researcher and contributors (e.g. changing roles, leave of absence for caregiving). Maintaining commitment over time was challenging; student lead may have improved this through more regular communication. | 29 |
| 5: Reflections / critical perspective | The SAG made substantial contributions to this project. As a solo student project, there were relatively few resources available to support the stakeholder engagement. In these circumstances, the following were crucial: a supervisor with substantial engagement experience, access to College Advisors, and a supportive employer.  RG’s background working as a part of multidisciplinary teams including patients was helpful, particularly given the constraints of the project, as was working with experienced and knowledgeable stakeholders. However, this limited the range of perspectives included. Notably, the SAG were also a valuable source of friendship and guidance, embodying the value of learning together so central to LHSs.  While engagement was not a requirement and, in fact, needed to be carefully negotiated around the degree requirements, it was a worthwhile investment that prioritized areas relevant to local policy audiences, enriched the findings, and is now helping to connect them to practice. | 29 |
