# Supplementary material for: The role of patients, caregivers, and communities in Learning Health Systems: a narrative review
Source: Front Health Serv. 2025 Oct 21;5:1606124. doi: 10.3389/frhs.2025.1606124 (PMC12584131; doi:10.3389/frhs.2025.1606124)
Supplement: Supplementary file 2 [file Supplementaryfile2.docx]

Supplementary Material 2. Sample Searches

**Initial search cycle**

Focus on identifying secondary research that synthesizes primary studies relevant to the research topic and/or protocols for planned evidence syntheses on the topic.

Databases searched on November 8, 2022:

- PROSPERO
- PubMed

(patient engagement) AND (Learning Health Systems) AND ((review[Filter] OR systematicreview[Filter]) AND (2007:2022[pdat]))

**Core search strategy**

- Structured database searches focused on key domains
- Search syntax initially developed for PubMED (below) and then translated for other databases.
- For search syntax translations and search results for CINAHL, PsycINFO, and EMBASE see supplemental data file: Supplementary-Material-3_Data-DB-Searches.xls.

**Sample search result: PubMED**

| **#** | **Query** | **Results** | **Date** | **Uploaded to Covidence** |
| --- | --- | --- | --- | --- |
| 1 | ((participation[tiab] OR participating[tiab]) AND (patient*[tiab] OR participant*[tiab] OR people[tiab] OR person*[tiab] OR client* [tiab] OR user* [tiab] OR advisor* [tiab] OR consumer* [tiab] OR caregiver* [tiab] OR family [tiab] OR families[tiab] OR relative* [tiab] OR community [tiab] OR communities [tiab] OR citizen* [tiab] OR public*[tiab] OR "lay person*" [tiab] OR "lay people" [tiab] OR "lay-person*" [tiab] OR "lay-people" [tiab] OR "People with Lived Experience" [tiab] OR "Experts by experience"[tiab])) | **182,787** | 06-Dec-22 | No |
| 2 | ((engagement[tiab] OR engage[tiab] OR engaging[tiab] OR engaged [tiab]) AND (patient*[tiab] OR participant*[tiab] OR people[tiab] OR person*[tiab] OR client* [tiab] OR user* [tiab] OR advisor* [tiab] OR consumer* [tiab] OR caregiver* [tiab] OR family [tiab] OR families[tiab] OR relative* [tiab] OR community [tiab] OR communities [tiab] OR citizen* [tiab] OR public*[tiab] OR "lay person*" [tiab] OR "lay people" OR [tiab] "lay-person*" [tiab] OR "lay-people" [tiab] OR "People with Lived Experience" [tiab] OR "Experts by experience"[tiab])) | **243** | 06-Dec-22 | No |
| 3 | ((involvement[tiab] OR involve[tiab] OR involving[tiab] OR involved[tiab]) AND (patient*[tiab] OR participant*[tiab] OR people[tiab] OR person*[tiab] OR client* [tiab] OR user* [tiab] OR advisor* [tiab] OR consumer* [tiab] OR caregiver* [tiab] OR family [tiab] OR relative* [tiab] OR families[tiab] OR community [tiab] OR communities [tiab] OR citizen* [tiab] OR public*[tiab] OR "People with Lived Experience" [tiab] OR "Experts by experience"[tiab])) | **1,091,511** | 06-Dec-22 | No |
| 4 | ((partnership[tiab] OR partner[tiab] OR partnering[tiab] OR partnered[tiab]) AND (patient*[tiab] OR participant*[tiab] OR people[tiab] OR person*[tiab] OR client* [tiab] OR user* [tiab] OR advisor* [tiab] OR consumer* [tiab] OR caregiver* [tiab] OR family [tiab] OR families[tiab] OR relative* [tiab] OR community [tiab] OR communities [tiab] OR citizen* [tiab] OR public*[tiab] OR "lay person*" [tiab] OR "lay people" [tiab] OR "lay-person*" [tiab] OR "lay-people" [tiab] OR "People with Lived Experience" [tiab] OR "Experts by experience"[tiab])) | **76,901** | 06-Dec-22 | No |
| 5 | ("patient-driven research"[tiab] OR "patient-led research"[tiab] OR "participatory action research"[tiab] OR "community-based participatory research"[tiab] OR "co-design*"[tiab] OR "codesign"[tiab] OR "co-production"[tiab] OR "evidence-based co-design"[tiab]) | **10,549** | 06-Dec-22 | No |
| 6 | "Patient Partner*" [tiab] OR "Patient and Family Advisor*"[tiab] OR "Essential Care Partner" [tiab] OR "Patient/Public Partner" [tiab] OR "Patient Partner*"[tiab] | **857** | 06-Dec-22 | No |
| 7 | Consumer Participation[mh:noexp] | **18,300** | 06-Dec-22 | No |
| 8 | Patient Participation[mh:noexp] | **29,036** | 06-Dec-22 | No |
| 9 | Social Participation[mh:noexp] | **3,308** | 06-Dec-22 | No |
| 10 | Community-Based Participatory Research[mh:noexp] | **5,463** | 06-Dec-22 | No |
| 11 | ("Learning Healthcare System*"[tiab] OR "Learning Health Care System*"[tiab] OR "Learning Health Organization*"[tiab] OR "Learning Health Organisation*"[tiab] OR "Learning Healthcare Organization*"[tiab] OR "Learning Healthcare Organisation*"[tiab] OR "Learning Health Care Organization*"[tiab] OR "Learning Health Care Organisation*"[tiab] OR "Learning Health System*"[tiab] OR "LHS"[tiab] OR "Learning Health Network*"[tiab] OR "Clinical Learning Network*"[tiab] OR "Rapid Learning System*"[tiab] OR Learning Health System[mh:noexp]) | **1,952** | 06-Dec-22 | No |
| 12 | #1 AND #11 | **108** | 06-Dec-22 | Yes |
| 13 | #2 AND #11 | **29** | 10-Dec-22 | Yes |
| 14 | #3 AND #11 | **133** | 06-Dec-22 | Yes |
| 15 | #4 AND #11 | **57** | 06-Dec-22 | Yes |
| 16 | #5 AND #11 | **24** | 06-Dec-22 | Yes |
| 17 | #6 AND #11 | **2** | 06-Dec-22 | Yes |
| 18 | #7 AND #11 | **6** | 06-Dec-22 | Yes |
| 19 | #8 AND 11 | **18** | 06-Dec-22 | Yes |
| 20 | #9 AND 11 | **0** | 06-Dec-22 | No |
| 22 | #10 AND #11 | **4** | 06-Dec-22 | Yes |
|  | **Total uploaded to COVIDENCE** | **381** |  |  |
|  | **DUPLICATES in PubMed** | **77** |  |  |
|  | **TOTAL NEW RECORDS IN COVIDENCE AFTER DE-DUPPING** | **313** |  |  |
