# Supplementary material for: The role of patients, caregivers, and communities in Learning Health Systems: a narrative review
Source: Front Health Serv. 2025 Oct 21;5:1606124. doi: 10.3389/frhs.2025.1606124 (PMC12584131; doi:10.3389/frhs.2025.1606124)
Supplement: Supplementary file 4 [file Supplementaryfile4.docx]

Supplementary Material 4. Learning Health Systems grouped by level of implementation

If a source reported on multiple LHSs, only LHSs that met inclusion criteria were included in the analysis. One source does not appear in this table because it reports collectively based on experiences from 16 LHSs (76).

| **Name** | **Country** | **Description** | **LHS Focus** | **Source(s)** |
| --- | --- | --- | --- | --- |
| **Meso-level LHSs** | | | | |
| **Cincinnati Children's Hospital (CCHMC)* - Diabetes Center**  **Also see ICN and ACT* | US | The Diabetes Center provides “care for nearly all children with type 1 diabetes (T1D) in the Greater Cincinnati region” (95, p. 3). | Specific disease | (95) |
| **Dartmouth Spine Center** | US | Limited detail provided. LHS using Dartmouth LHS Model of Coproduction (105). | Specific disease | (105) |
| **Geisinger Health** | US | Large health organization comprising “13 hospital campuses, an addiction treatment center, a 550,000-member health plan, a medical school, and other facilities, staffed by over 30,000 employees, including 1800 physicians…serves 3 million residents, and provides care to approximately 1 million patients annually” (86, p. 3). | Healthcare organization | (86) |
| **IWK Health** | Canada | “Canadian tertiary health centre serving children, youth, and women from the four Atlantic Canadian provinces” (18, p. 3). | Specific population;  Healthcare organization | (18) |
| **Learning Health Care System Platform** | US | Limited detail provided. LHS based at Vanderbilt University Medical Center (VUMC). | Healthcare organization | (82) |
| **Promise Partnership Oncology LHS** | US | Unnamed “comprehensive cancer care center in Northern New England” that cares for roughly “32,000 patients annually and includes 17 interdisciplinary clinical oncology groups” (96, p. 2). | Specific disease | (96) |
| **Reducing Cancer Disparities by Engaging Stakeholders (RCaDES)** | US | LHS focused on cancer screening and implemented at Jefferson Health (JH), a large regional health system serving 1,560,000 people, and Lehigh Valley Health Network (LVHN) region serving 800,000 people. | Specific disease | (93) |
| **Registry-enabled care and learning system for cystic fibrosis (RCLS-CF)** | Sweden  US | Main team is based at the Dartmouth Institute for Health Policy & Clinical Practice in the USA. Support from the US Cystic Fibrosis Foundation, and advice from Cincinnati Children’s Hospital Medical Center (U.S.A.) and the Karolinska Institutet (Sweden). | Specific disease | (104) |
| **Ryhov Hospital - Self-Dialysis Unit** | Sweden | Self-dialysis unit “serves a local population of 145,000 and offers regional services for the county population of 340,000 citizens " (99, p. 4). | Specific disease | (77,99) |
| **University of Wisconsin (UW) Health** | US | Academic health centre comprising “6 hospitals, 90 regionally based clinics, and a physician practice plan. The 1400‐member faculty physician practice group provides care during ~2.4 million outpatient visits and ~28,000 hospitalizations per year at the university hospital and trains more than 550 residents and fellows across 60 accredited programs…Nearly 400 primary care providers care for 360,000 medically homed patients at over 40 different clinic practice locations” (94, p. 2). | Healthcare organization | (94) |
| **Wake Forest University School of Medicine / Wake Forest Baptist Health (WFSM/WFBH)** | US | WFUSM/WFBH comprises WFSM and 5 hospitals serving more than 50 counties in North Carolina, Tennessee, Virginia, and West Virginia. It has also established a Clinical and Translational Science Institute (CTSI) at WFUSM. | Healthcare organization | (75,101) |
| **Large networks** | | | | |
| **All Children Thrive Learning Network (ACT)** | US | An LHS started by Cincinnati Children's Hospital (CCHMC) that “convenes [quality] improvement teams including members of the healthcare system, community organizations, the public school system, and parents from the community” (95, p. 7). | Specific population | (95) |
| **Alliance for Healthier Communities** | Canada | A network supporting more than 100 “community-governed primary healthcare organizations in Ontario including Community Health Centres (CHCs), Aboriginal Health Access Centres, Community Family Health Teams and Nurse Practitioner Led Clinics” (85, p. 2). | Specific population; Level of care | (85) |
| **Alliance of Chicago Community Health Services** | US | A “network of community health centers serving primarily low-income and uninsured patients” (81, p. 125). | Specific population | (81) |
| **Autism Speaks Autism Treatment Network (ATN), Autism Intervention Research Network on Physical Health (AIR-P)** | US^[[1]](#footnote-1)^ | Network of “12 academic medical centres” that “provides clinical services to over 35,000 children with ASD [autism spectrum disorder] annually” (88, p. 1-2). | Specific disease | (79,88) |
| **Collaborative Chronic Care Network (C3N)** | US | A network that focuses on pediatric inflammatory bowel disease (IBD) and includes “24 care centers, with data on 2500 patients from 7500+ visits " (90, p. 2). | Specific disease | (90) |
| **Compartive Effectiveness** **Research Translation Network (CERTAIN)** | US | Network within Washington State Surgical Care and Outcomes Assessment Program (SCOAP) that involves “55 of 60 hospitals (92%) in Washington State” (72, p. S123). Initial focus on peripheral artery disease. | Specific disease; Specific population | (72) |
| **Connected Health Cities** | UK | Not provided  *Details available in Steels et al.* (139,140) | Not provided | (15) |
| **Electronic Data Methods Forum** | US | Network involving more than 70 institutions in discrete projects that “represent at least 21 million patients across the USA, and reflect an unprecedented mix of patient types (e.g. pediatric, adult, elderly, low income, disabled), conditions (e.g. asthma, diabetes, hypertension) and care settings (inpatient, outpatient, specialty clinics) ” (83, p. 636). | Infrastructure | (83) |
| **ImproveCareNow (ICN)** | US  Belgium  Qatar  UK | Providing care for roughly 30,400 pediatric inflammatory bowel disease (IBD) patients at more than 100 care centres int the USA, Belgium, Qatar and UK. | Specific disease | (68,74,81,90,  99,105) |
| **patient-centered Scalable National Network for Effectiveness Research (pSCANNER)** | US | Participating network members include academic medical centres and health care clinics serving a diverse patient population of more than 22 million people. | Infrastructure | (89) |
| **Pediatric Rheumatology Care and Outcomes Improvement Network (PR-COIN)** | US  Canada | Involves 17 care centers in the U.S.A. and Canada, and aims to improve outcomes for children and youth with pediatric rheumatic diseases. | Specific disease | (92) |
| **Starzl Network for Excellence in Pediatric Transplantation (SNEPT)** | US  Canada | Multicentre network of tertiary care facilities performing pediatric liver transplantations. | Specific disease | (87,91) |
| **Publicly-funded, macro-level LHSs** | | | | |
| **Early Intervention Services for Psychosis in Québec** | Canada | LHS approach to providing early intervention services for people with psychotic disorders. | Specific disease; Provincial health system | (103) |
| **Long Covid Multidisciplinary consortium Optimising Treatments and servIces cross the NHS (LOCOMOTION)** | UK | Led by University of Leeds, LOCOMOTION engages a large network of academic collaborators including National Health Service (NHS) long COVID clinics. | Specific disease; National health system | (98) |
| **Northwest Territories Health System** | Canada | Publicly-funded care to people in Northwest Territories (NT), of which “50.2% self-identifies as Indigenous, a group that comprises First Nations, Inuit, and Metis " (97, p. 3-4). In partnership with Gwich’in Tribal Council and Tłı̨chǫ Government, and Government of Northwest Territories. | Provincial health system | (97) |
| **Post COVID-19 Interdisciplinary Clinical Care Network (PC-ICCN)** | Canada | Provides care to a “subset of post COVID-19 patients with persistent symptoms” through specialized clinics…“in 950,000 km^2^ area with estimated population of 5 million people” (84, p. 2). | Specific disease; Provincial health system | (84) |
| **Saskatchewan Provincial Health System** | Canada | Not provided. | Provincial health system | (80) |
| **Sweden’s National Healthcare System** | Sweden | "Sweden's universal health system" which is “nationally regulated and locally provided by 21 regions and 290 municipalities" (78). | National health system | (78) |
| **U.S. Veterans Affairs (VA)** | US | Large LHS providing care to more than "2 million military servicemembers, 20 million veterans, and millions more military and veteran family members” (102, p. 631). Several LHSs are nested within the VA including STRONG STAR Consortium (102), Veteran Affairs Evidence Synthesis Program (ESP) (73), pSCANNER (89). | Specific population; National health system | (73,89,102) |

1. One source indicated that the network operates in North America (88). [↑](#footnote-ref-1)
